# TNF-α and Poly(I:C) induction of A20 and activation of NF-κB signaling are independent of ABCF1 in human airway epithelial cells

**DOI:** 10.1038/s41598-023-41990-w

**Published:** 2023-09-07

**Authors:** Quynh T. Cao, Mira Ishak, Israel Shpilman, Jeremy A. Hirota

**Affiliations:** 1 Department of Medicine, McMaster University, Hamilton, ON Canada; 2 Division of Respirology, Department of Medicine, Firestone Institute for Respiratory Health, McMaster University, Hamilton, ON L8N 4A6 Canada; 3 Department of Biology, University of Waterloo, Waterloo, ON Canada; 4 Division of Respiratory Medicine, Department of Medicine, University of British Columbia, Vancouver, BC V6H 3Z6 Canada

**Keywords:** Cytokines, Innate immunity, Immunology, Mucosal immunology

## Abstract

ABCF1 is the most characterized member of the ABCF family in eukaryotes with proposed functions related to innate immunity in fibroblasts, macrophages, and epithelial cells. Currently, a mechanistic link between ABCF1 and immune responses in human airway epithelial cells (HAECs) remains to be clearly defined. The present study aimed at characterizing the function of ABCF1 in the context of nuclear factor nuclear factor κB (NF-κB) mediated pro-inflammatory responses in an immortalized human airway epithelial cell line, HBEC-6KT. We demonstrated that with ABCF1 silencing under basal conditions, TNF Alpha Induced Protein 3 (TNFAIP3/A20) protein expression and downstream expression and activation of transcription factors, NF-κB and Interferon regulatory factor 3 (IRF-3), were not disrupted. We followed with investigations of ABCF1 function under a pro-inflammatory stimuli that are known to be regulated by A20. We demonstrated that under Polyinosinic:polycytidylic acid (Poly(I:C)) and tumor Necrosis Factor-α (TNF-α) challenge with ABCF1 silencing, there was a significant reduction in secreted levels of interleukin-8 (IL-8) and a trend for reduced IL-6. However, we observed no changes to the expression levels of A20 and the activation status of the transcription factors, NF-κB and IRF-3. Collectively, these studies demonstrate that Poly(I:C) and TNF-α induced IL-8 is regulated by ABCF1 via pathways independent of NF-κB and IRF-3 activation.

## Introduction

The pseudostratified airway epithelium is a barrier that protects the lung from the external environment and is essential for providing defence against pathogens and airborne particles that can cause acute and chronic injuries to the lungs^1^. The airway epithelium has evolved to protect the lungs against these challenges by providing a tight barrier formed by apical tight junctions and adherens junctions, in addition to a mucociliary escalator that helps clear inhaled substances trapped in mucus that lines the airways^1^. In addition to forming a physical barrier, airway epithelial cells express an array of pattern recognition receptors, including toll-like receptors (TLRs), C-type lectin receptors (CLRs), retinoic acid-inducible gene I-like receptors (RLRs), and NOD-like receptors that can recognize respiratory pathogens to coordinate an appropriate immune response designed to protect the host^1^. These responses include producing soluble factors such as antimicrobial peptides (AMPs), cytokines, chemokines, and growth factors to recruit leukocytes, modulate the adaptive immune system, and initiate tissue repair^1^.

ATP Binding Cassette (ABC) Transporter family of proteins are conserved in prokaryotes and eukaryotes. Most ABC transporters function through the coupling and hydrolysis of ATP to allow for extracellular transport of substrates including cytokines, ions, lipids, and xenobiotics. To date, 49 known human ABC transporters can be divided into 7 subclasses (ABCA, ABCB, ABCC, ABCD, ABCE, ABCF, and ABCG)^2^. Some ABC transporters have been reported to have major implications in lung health and disease including ABCC7, more commonly known as cystic fibrosis transmembrane conductance regulator (CFTR) that is involved in regulating chloride and bicarbonate transport; ABCA3 is important for surfactant production; and ABCC4, a modulator of anti-inflammatory activities^3–5^. The ABCF family are unique in their structure and function as they lack a transmembrane domain and therefore lack the capacity for transporting substrates^6^. ABCF1 is the most characterized member of the ABCF family in eukaryotes with a wide range of proposed functions depending on the cell types, tissues, and species.

The earliest studies have characterized ABCF1 as being involved in protein translation through its interactions with eIF2 and ribosomal proteins, but it was not specified under what conditions or what types of protein expression it regulates^7–9^. Other studies have demonstrated its importance in embryonic development through its roles in gene transcription and translation. It was demonstrated that *Abcf1* knockout mice models are embryonically lethal because ABCF1 is involved in actively proliferating and differentiating cells during embryo development^10^. A separate group has demonstrated that ABCF1 is required for stem cell self-renewal as it acts as a co-activator for OCT4 and SOX2^11^. Regarding immune activities, one study has showed that ABCF1 acts as a cytosolic sensor to viral dsDNA in mouse embryonic fibroblasts, and another study showed that ABCF1 acts as an E2 ubiquitin-conjugating enzyme that controls macrophage polarization^12, 13^.

Our group have confirmed the expression of ABCF1 in human airway epithelial cells (HAEC) at the gene and protein level both in situ and in vitro in our previous studies. In addition, we characterized the function of ABCF1 as an important component in modulating innate immune responses in HAECs. We used a hypothesis-free gene ontology (GO) analysis and identified the key pathways that were impacted by ABCF1 siRNA treatment during a cytosolic nucleic acid challenge^14^. One of these pathways included TLR-3/4 signalling and the key gene that was differentially expressed in these pathways was *TNFAIP3*. This gene is expressed as the A20/tumor necrosis factor α-induced protein 3 (TNFAIP3) protein, a ubiquitin ligase and deubiquitinating enzyme that inhibits nuclear factor κB (NF-κB) and interferon regulatory factor 3 (IRF-3) activation to regulate innate immune responses^15, 16^. To our knowledge, a mechanistic link between ABCF1 expression and A20, NF-κB, and IRF-3 signaling has not been explored in HAECs.

Previously, stimulation of synoviocytes with Tumor Necrosis Factor α (TNF-α) demonstrated a potential role of ABCF1 in promoting inflammatory responses. The study linked pro-inflammatory challenge in vitro with the mRNA transcript of *Abcf1* accumulating in synoviocytes, without mechanistically linking this relationship to functional responses^17^. In macrophages, ABCF1 was shown to be regulating MyD88- and TRIF-dependent pro-inflammatory signalling in response to lipopolysaccharide (LPS) challenge. It was demonstrated that by silencing of ABCF1, it led to a decrease in protein expression of A20, an increase in NF-κB and a decrease in IRF-3 activation under basal and LPS challenged conditions. This finding suggested that ABCF1 could be negatively regulating the MyD88-dependent signalling and positively regulate the TRIF-dependent signalling in response to LPS challenge^13^. Currently, there are no known implications for lung health and disease associated with ABCF1. Nonetheless, we are intrigued by the possibility of exploring whether ABCF1 plays a functional role in the airway epithelium and whether it has an influence on lung health like certain members of the ABC transporter family of proteins. The two studies mentioned provides an associative and mechanistic links between ABCF1 with immune responses, thereby supporting the need for a more comprehensive exploration ABCF1’s characterization in HAECs.

Building upon the conclusions drawn from our earlier study, we put forth the hypothesis that suppressing ABCF1 in HBECT-6KT HAECs will result in a reduction of A20 protein expression, thereby intensifying the pro-inflammatory reactions mediated by NF-κB. In this study, we performed mechanistic studies with ABCF1 silencing under basal and stimulated conditions with readouts of A20, NF-κB, and IRF-3 expression and activation. We demonstrated that under basal unstimulated conditions, ABCF1 knockdown does not impact A20 protein expression or NF-κB and IRF-3 activation. Furthermore, despite the silencing of ABCF1 leading to attenuation of Polyinosinic:polycytidylic acid (Poly(I:C)) and TNF-α induced IL-8, no changes were observed in A20 protein expression or NF-κB and IRF-3 activation. Our results demonstrate that in HBEC-6KT HAECs, ABCF1 regulates IL-8 induction independent of canonical pro-inflammatory pathways and suggests novel regulatory mechanisms for this cytokine.

## Methods

### Reagents

ABCF1, A20, NF-κB p65, phosphorylated NF-κB p65 (Ser536), IRF-3 and phosphorylated IRF-3 (Ser386) were probed with anti-ABCF1 primary antibody (HPA017578, Sigma-Aldrich, Burlington, Massachusetts) at 1:500–1:1000, anti-A20 primary antibody (5630T, Cell Signaling Technology, Danvers, Massachusetts) at 1:1000, anti-NF*-*κB p65 primary antibody (4764T, Cell Signaling Technology) at 1:1000, anti-NF*-*κB p65 (Ser536) primary antibody (3033T, Cell Signaling Technology) at 1:1000, anti-IRF-3 (ab76493, Abcam, Waltham, Boston) at 1:1000 and anti-IRF-3 (Ser386) (ab68481, Abcam) at 1:1000 in 3% Casein (1706404, Bio-Rad Laboratories, Hercules, California) in 1X Tris Buffered Saline (T5912, Sigma-Aldrich) with TWEEN^®^ 20 (P1379, Sigma-Aldrich) (TBS-T). The secondary antibody used for each protein was anti-rabbit HRP-Linked Antibody from Cell Signaling Technology (7074S, Cell Signaling Technology) at 1:2000 in 3% Casein in TBS-T. ABCF1 (si-ABCF1) and scramble siRNA (siCtrl) SMARTpool siGENOME, as well as the DharmaFECT 1 transfection reagent were purchased from Dharmacon (M-008263-01 and D-001206-14, Lafayette, Colorado). The SMARTpool siRNA reagent is a pool of 4 siRNA duplexes all designed to target distinct sites within the specific gene of interest. The 4 different siRNA within the pool were selected by Dharmacon using their design algorithm to have the optimal silencing of the target transcript NM_001025091 and NM_001090, with all siRNA targeting within the open reading frame. The immunostimulatory challenges, TNF-α was purchased from Peprotech (300-01A, Rocky Hill, New Jersey), Recombinant human IL-17A protein was purchased from R&D Systems (7955-IL-025, Minneapolis, Minnesota); TLR-3 Agonist (tlrl-pic), TLR-4 Agonist (tlrl-b5lps), and TLR-2 Agonist (tlrl-pgns2) were all purchased from Invivogen (San Diego, California). All challenges were directly added to the cells with cell culture media without a transfection reagent.

### Cell culture

All experiments were performed in submerged monolayer cell culture using the HBEC-6KT immortalized HAEC line over expressing human telomerase reverse transcriptase (hTERT) and cyclin-dependent kinase 4 (Cdk4)^18–22^. The cell line was obtained from lung biopsies that were not histologically involved with lung cancer from non-smoker donors and it does not have a malignant phenotype^18^. HBEC-6KT were grown in keratinocyte serum free medium (ThermoFisher Scientific, Waltham, Massachusetts) supplemented with 0.8 ng/ml epithelial growth factor, 50 µg/ml bovine pituitary extract and 1 X penicillin/streptomycin (97,063-708, VWR, Radnor, Pennsylvania). All cells were grown at 37 °C at 5% CO_2_.

### In vitro pro-inflammatory challenge experiments with siRNA-mediated knockdown of ABCF1

Pro-inflammatory dose response experiments were performed in HBEC-6KT cells with immunostimulatory challenges for 24 hours (h) at a confluency of approximately 80–90%. Cells were challenged with TNF-${\upalpha }$ (10–1000 ng/ml), Poly(I:C) (0.01–1 μg/ml), PGN-SA (1–100 μg/ml), IL-17A (0.1–10 ng/ml), and LPS-B5 (0.01–1 μg/ml).

**Figure 1:**
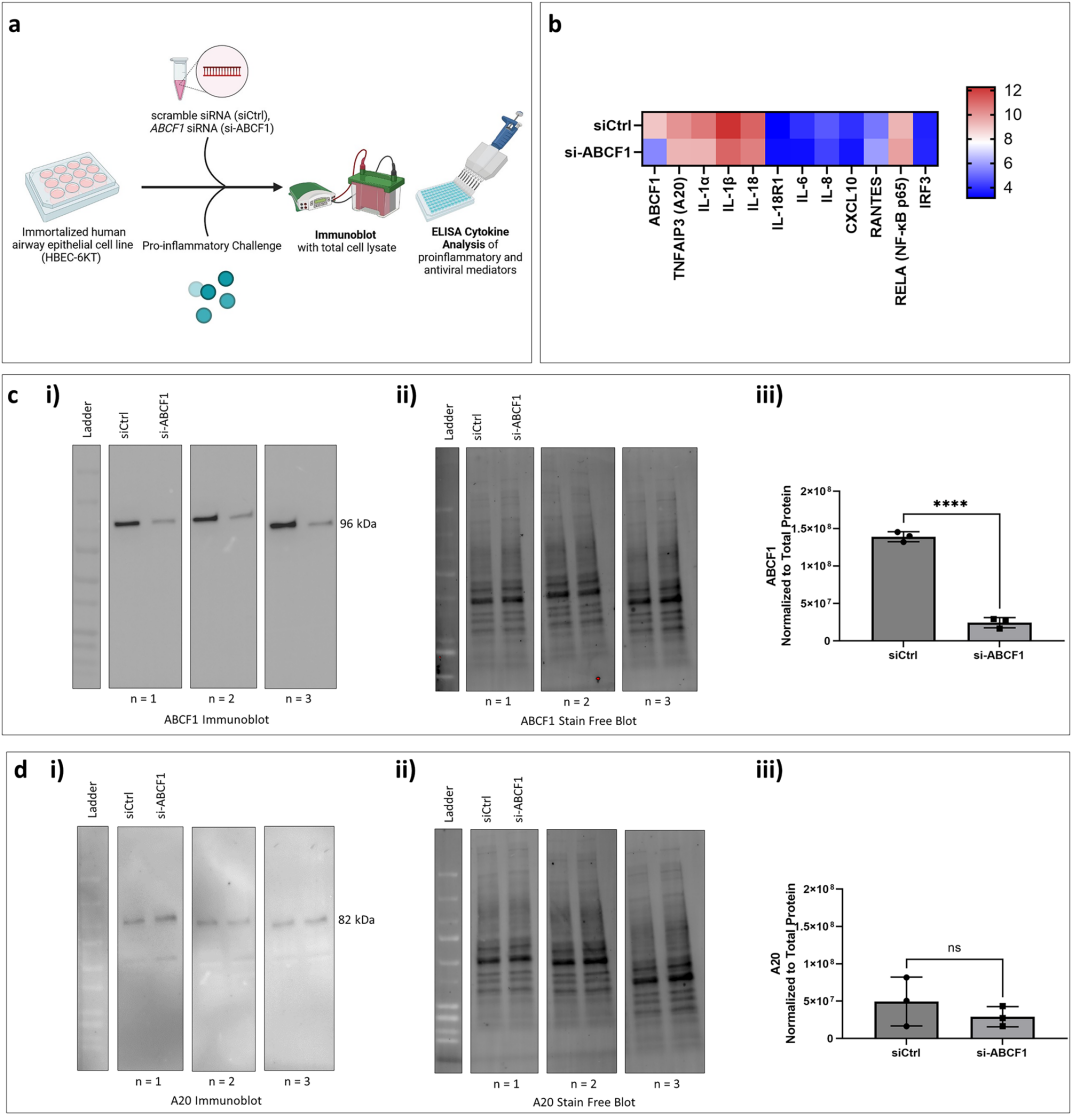
ABCF1 silencing on A20 protein expression under basal conditions in HBEC-6KT in vitro. (**a**) Experimental design schematic. Immortalized human airway epithelial cells grow in cell culture plates, the undergoes ABCF1 siRNA-mediated knockdown followed by pro-inflammatory challenge. Total cell lysates collected from the cells are used for immunoblotting and cell culture supernatant are used to run ELISAs. (**b**) Heat map of log_2_ expression data of select genes associated with antiviral and pro-inflammatory responses for VACV-70 samples with and without si-ABCF1. (**c)** (**i**) Immunoblot to confirm siRNA-mediated knockdown of ABCF1 protein expression in HBEC-6KT cells under basal conditions. (**ii**) Stain-free blot was used to (**iii**) quantify ABCF1 protein expression. (**d**) (**i**) Immunoblot to confirm siRNA-mediated knockdown of A20 protein expression in HBEC-6KT cells under basal conditions. (**ii**) Stain-free blot was used to (**iii**) quantify ABCF1 protein expression. All studies n = 3; ****p < 0.0001.

All in vitro siRNA-mediated knockdown experiments in HBEC-6KT were done using siRNA transfected with DharmaFECT Transfection Reagent according to the manufacturer’s instructions. Cells were transfected with si-ABCF1 or siCtrl for 24 h at approximately 70% confluency. After siRNA-mediated knockdown when the cells are at 80–90% confluency, the cells were challenged for 24 h followed by outcome measurements of function (cytokine secretion measured by ELISA) and protein expression (immunoblot) (Fig. 1a). Similar experiments were performed in the absence of challenges to investigate baseline ABCF1 function.

### Cytokine analysis

Cell culture media were collected following in vitro experiments and centrifuged at 7500×*g* for 12 min. ELISA assays were run for human IL-6 (DY206, R&D Systems, Minneapolis, Minnesota) and IL-8 (DY208, R&D Systems) according to manufacturer directions with absorbance read using the SpectraMax i3 Multi-Mode Platform (Molecular Devices, Silicon Valley, California) microplate reader. Cytokine analyses were performed with n = 5 and analyzed as unpaired student t-tests.

### Western blotting

HAECs were isolated using a cell scraper (83.395, Sarstedt, Nümbrecht, Germany) and lysed with RIPA lysis buffer (89900, ThermoFisher Scientific) containing protease inhibitor cocktail (P2714-1BTL, Sigma-Aldrich) and PhosSTOP (4906845001, Roche, Mississauga, Ontario). The collected protein was used for western blotting without further sample preparation. Protein from total cell lysate (4 μg) was premixed in 1X Laemmli Sample Buffer (1610747, Bio-Rad Laboratories), then separated on a 4–20% Mini-PROTEAN TGX stain-free precast gels (4568093, Bio-Rad Laboratories) and transferred to a PVDF membrane using the Transfer-Blot Turbo RTA Transfer Kit reagents (1704272, Bio-Rad Laboratories). Membranes were blocked at room temperature for 1 h using 5% Casein (1706404, Bio-Rad Laboratories) in 1X Tris Buffered Saline with TWEEN^®^ 20. Protein detection was performed using the Clarity Western ECL Substrate (1705061, Bio-Rad Laboratories) and imaged in a ChemiDoc MP Imaging System. All images were acquired using the auto-exposure setting. Signal intensity was normalized to total protein loading from membranes stained for total protein, on ImageLab (Bio-Rad Laboratories), a method that ensures normalization of protein signal is performed in the linear range of detection^23^. Western blot analyses were performed with n = 3 and analyzed as unpaired student t-tests. Although an n = 5 was performed for each experiment, an n = 3 was used for western blot analyses due to limited sample availability for all the blots required.

### Statistical analyses

The statistical analyses of the processed microarray data were performed following the same methodology as described in our previous study^14^. Determination of statistically significant differential gene expression was performed using empirical Bayes method via the eBayes function from limma R package. Gene expression analysis was conducted with a sample size of n = 3, and a gene-level *p*-value of < 0.05 was considered statistically significant.

All subsequent experiments were conducted with a minimum sample size of n ≥ 3, unless stated otherwise. Each repetition was obtained from a distinct cell culture. Experiments conducted with HBEC-6KT were considered independent when separated by at least one passage, all within a maximum of 5 passages. Statistical analysis was conducted using Ordinary One-Way ANOVA with a Dunnett’s test and unpaired student t-test comparing selected groups with p-value of < 0.05 determined to be statistically significant on GraphPad Prism 9.

## Results

### ABCF1 Knockdown does not impact A20 protein expression and function under basal conditions

We have previously performed a transcriptomic profiling with Gene Ontology (GO) term analysis on ABCF1 knockdown in response to VACV-70, dsDNA viral mimic challenge. Top-ranking GO pathway terms included *Regulation of toll-like receptor 3–4 signaling pathways*, driven by the genes *WDFY1*, *TNFAIP3* (A20) and *NR1D1*. Induction of the TNFR1 and TLR-3 signaling pathways can induce an innate immune response through NF-κB signaling pathway in HAEC. The gene *TNFAIP3*, expressed as A20 protein, is a master regulator of NF-κB activity by ubiquitination^15^. To follow up with our previous findings, we first investigated the expression and function of A20 in our model system under basal conditions.

First, we looked at the differential gene expression of A20, NF-κB p65 and NF-κB regulated cytokines and chemokines (Fig. 1b) under basal unstimulated conditions. Under siRNA-mediated knockdown of ABCF1, we observed a non-significant trend for downregulated gene expression of A20, IL-18, IL-6, IL-8, IL-α, IL-1β and CXCL10; and a non-significant trend for upregulated gene expression of NF-κB p65 and RANTES when compared to the siCtrl.

Next, we wanted to confirm A20, NF-κB p65, phosphorylated NF-κB p65 (p- NF-κB p65), IRF-3 and phosphorylated IRF-3 (p-IRF-3) protein expression with and without ABCF1 knockdown under basal unstimulated conditions using an immunoblot. We demonstrate significant ABCF1 knockdown of approximately 82.4% (Fig. 1c). Under this level of ABCF1 attenuation, baseline A20 protein expression (Fig. 1d) was not impacted.

**Figure 2:**
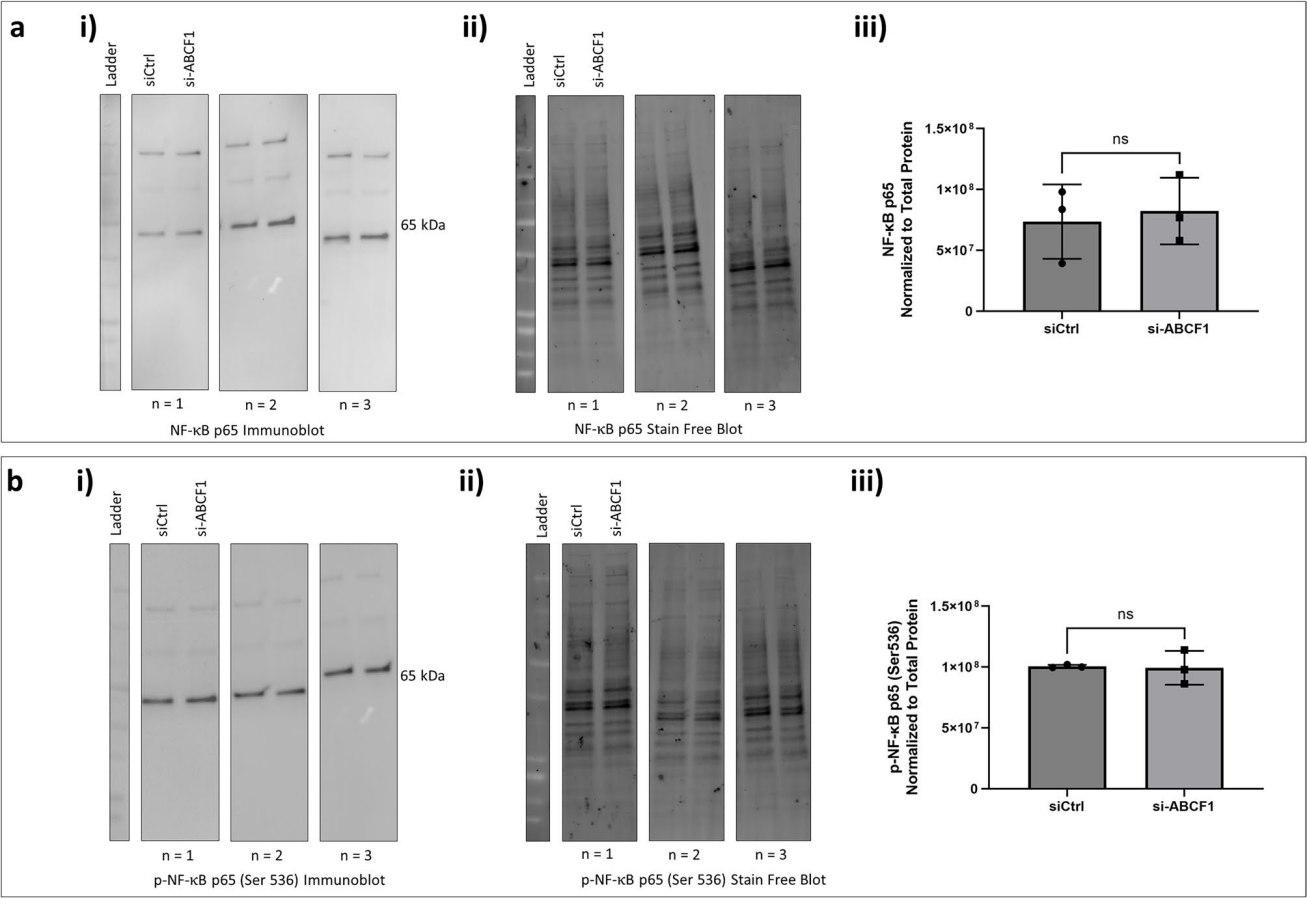
ABCF1 silencing on NF-κB p65 protein expression and activation under basal unstimulated condition in HBEC-6KT in vitro. (**a**) (i) Immunoblot of total NF-κB p65 protein expression with and without si-ABCF1 in HBEC-6KT cells under basal conditions. (**ii**) Stain-free blot was used to (**iii**) quantify NF-κB p65 protein expression. (**b**) (**i**) Immunoblot of phosphorylated NF-κB p65 (Ser 536) with and without si-ABCF1 in HBEC-6KT cells under basal conditions. (**ii**) Stain-free blot was used to (**iii**) quantify NF-κB p65 phosphorylation. All studies n = 3; ns > 0.05.

Upstream of A20 protein regulation is NF-κB signaling^15^. We therefore explored if NF-κB expression and activation were disrupted under basal unstimulated conditions with ABCF1 silencing. The protein expression of total NF-κB p65 (Fig. 2a) and its phosphorylation form (Fig. 2b) remained unchanged when compared to the control.

**Figure 3:**
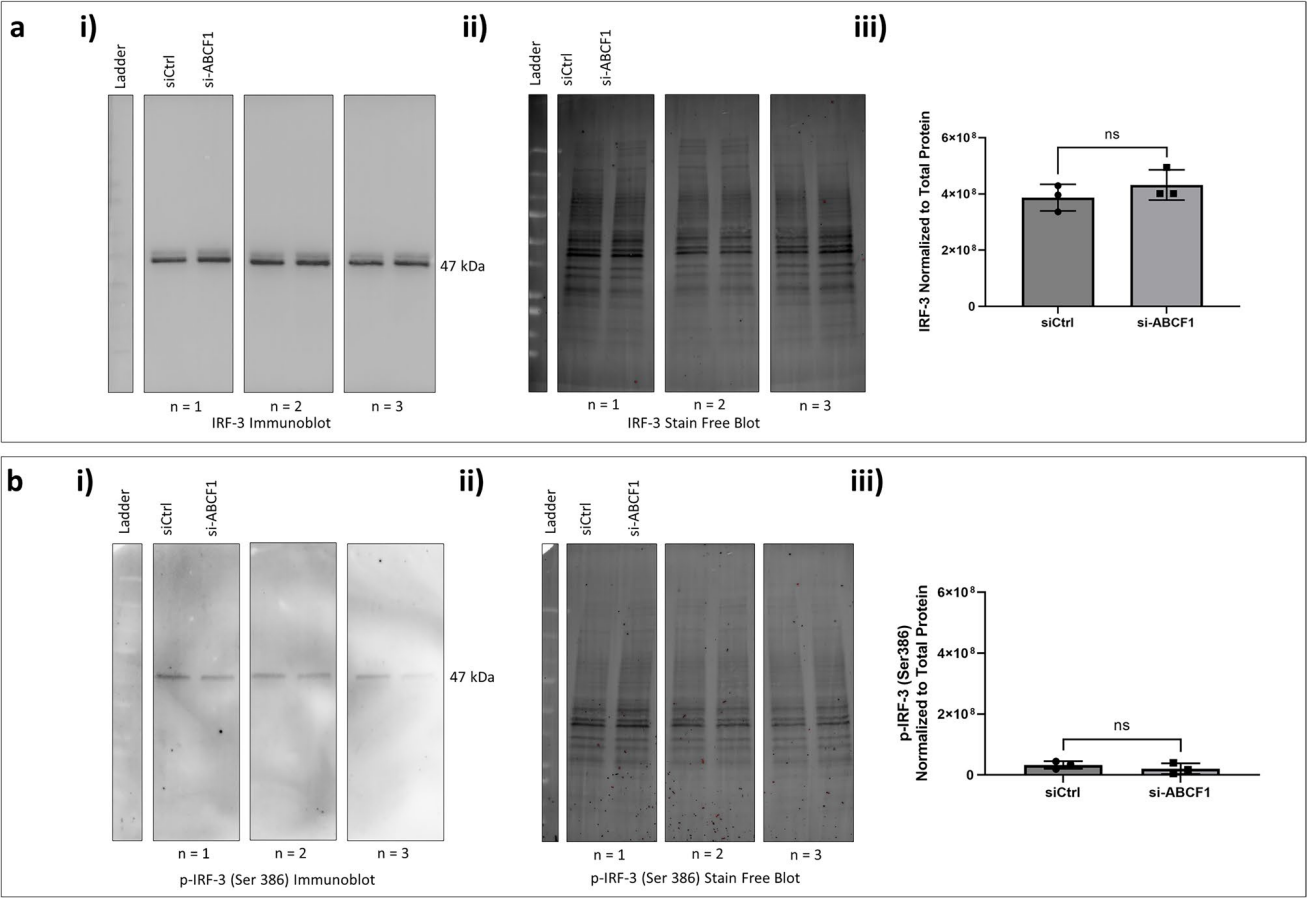
ABCF1 silencing on IRF-3 protein expression and activation under basal unstimulated condition in HBEC-6KT in vitro. (**a**) (**i**) Immunoblot of total IRF-3 protein expression with and without si-ABCF1 in HBEC-6KT cells under basal conditions. (**ii**) Stain-free blot was used to (**iii**) quantify IRF-3 protein expression. (**b**) Immunoblot of phosphorylated IRF-3 (Ser 386) with and without si-ABCF1 in HBEC-6KT cells under basal conditions. (**ii**) Stain-free blot was used to (**iii**) quantify IRF-3 phosphorylation. All studies n = 3; ns > 0.05.

In addition to A20 regulation, NF-κB signaling is also able to regulate IRF-3 signaling^16^. We therefore explored the protein expression of total IRF-3 and its phosphorylation. Similar to NF-κB, total IRF-3 (Fig. 3a) and phosphorylated form (Fig. 3b**)** were not impacted by ABCF1 knockdown.

Collectively, these data suggest that under basal unstimulated conditions, a reduction of ABCF1 greater than 80% does not impact A20, NF-κB and IRF-3 protein expression and activity in HAECs.

### Immunostimulatory challenges to induce A20 pro-inflammatory mediated responses in HAECs

Our experiments with HAECs did not reveal an obvious role for ABCF1 under basal unstimulated conditions. We therefore investigated the impact of ABCF1 on A20, NF-κB, and IRF-3 under pro-inflammatory challenges.

**Figure 4:**
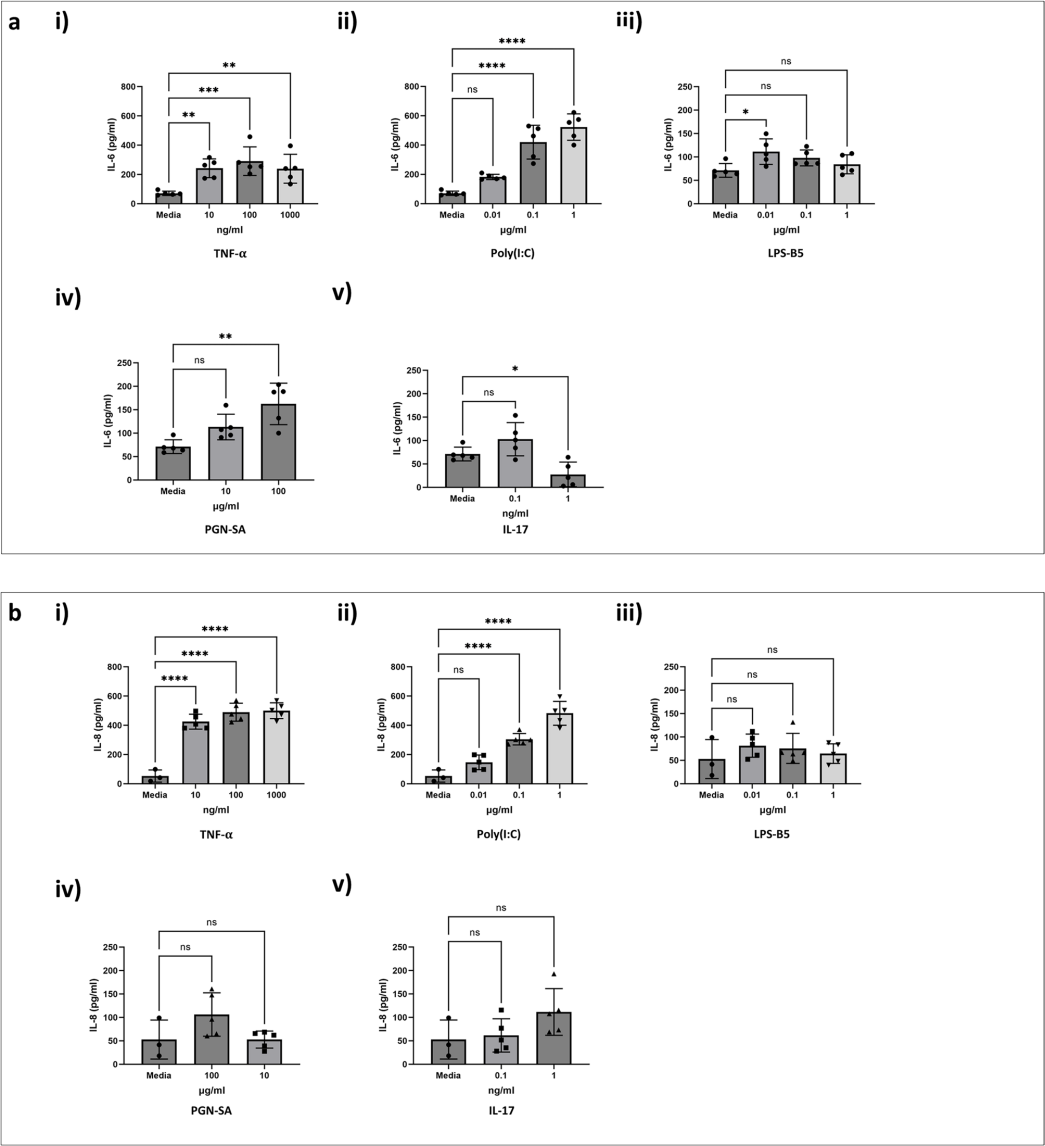
Immunostimulatory challenges induced responses in HBEC-6KT in vitro. (**a**) IL-6 and (**b**) IL-8 protein production in HBEC-6KT cells under concentration response induced by (**i**) TNF-${\upalpha }$, (**ii**) Poly(I:C), (**iii**) LPS-B5, (**iv**) PGN-SA, and (**v**) IL-17. All studies n = 3–5; **** *p* $\le$ 0.0001.

**Figure 5:**
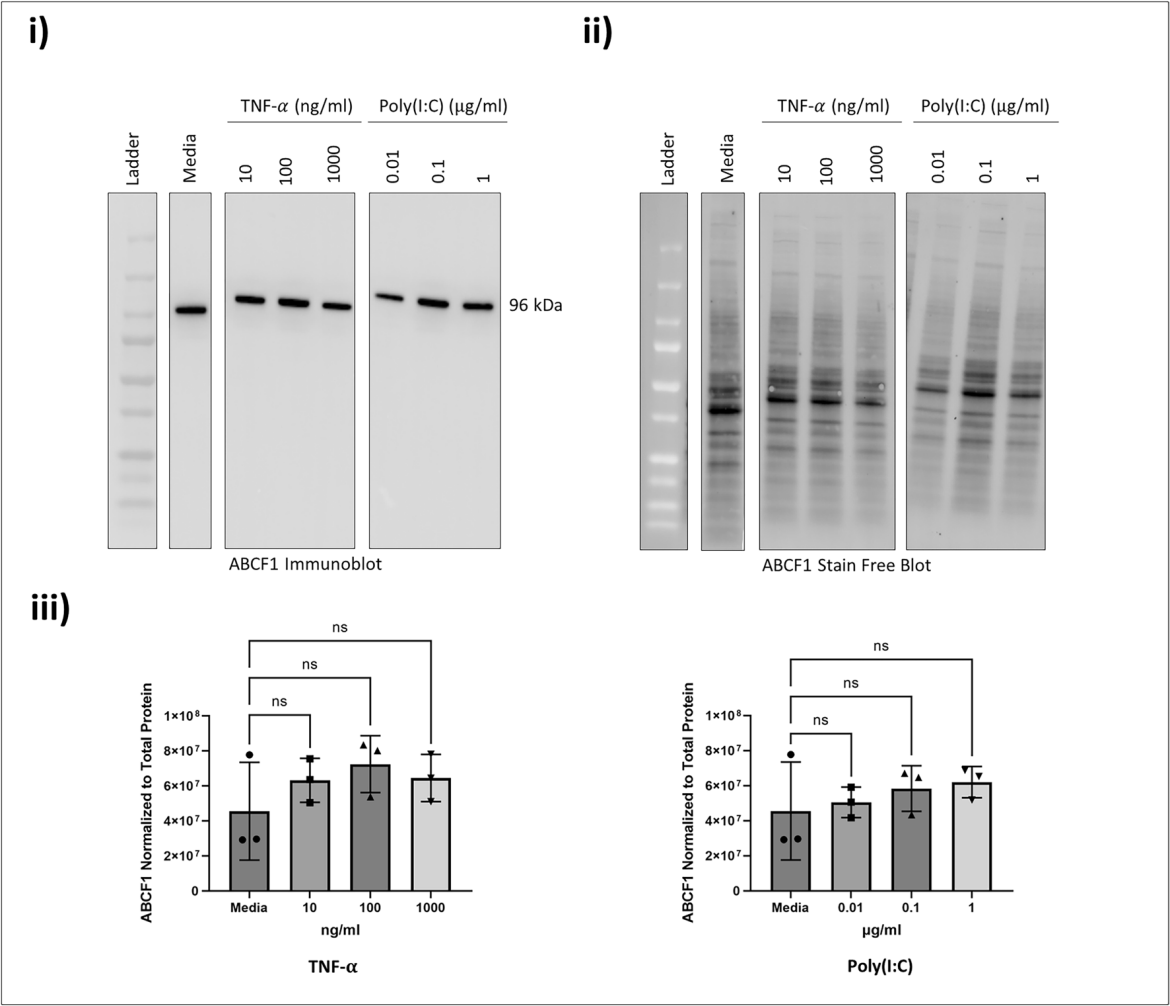
Immunostimulatory challenges on ABCF1 expression in HBEC-6KT in vitro. (**i**) Immunoblot of ABCF1 protein expression with TNF-α and Poly(I:C) dosage-based stimulation in HBEC-6KT cells. (**ii**) Stain-free blot was used to (**iii**) quantify ABCF1 protein expression. All studies n = 3; ns > 0.05.

**Figure 6:**
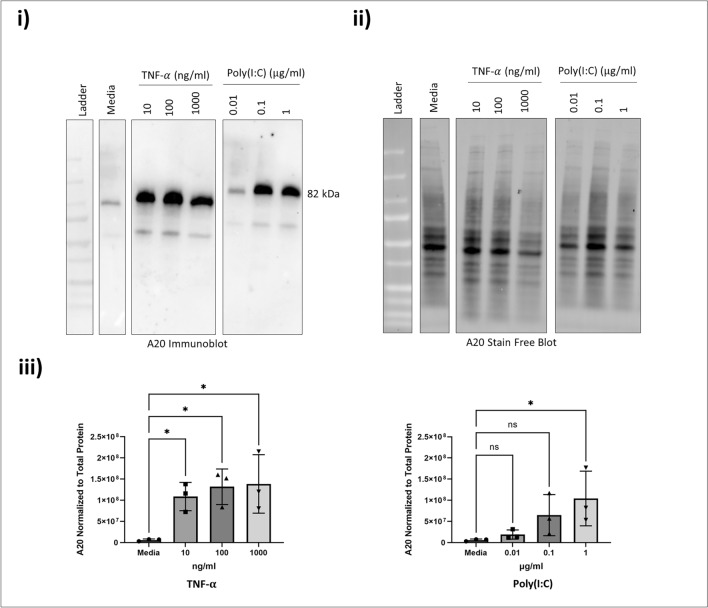
Immunostimulatory challenges on A20 protein expression in HBEC-6KT in vitro. (**i**) Immunoblot of A20 protein expression with TNF-α and Poly(I:C) dosage-based stimulation in HBEC-6KT cells. (**ii**) Stain-free blot was used to (**iii**) quantify A20 protein expression. All studies n = 3; ns > 0.05.

To select the appropriate challenges that can induce a pro-inflammatory response in HAECs, we selected five immunostimulatory challenges known to induce A20 mediated IL-6 and IL-8 responses^24–28^. We performed in vitro challenges at log concentrations with TNF-α (10–1000 ng/ml), IL-17 (0.1 to 10 ng/ml), LPS-B5 (0.01–1 μg/ml), PGN-SA (1–100 μg/ml), and Poly(I:C) (0.01–1 μg/ml). Following the challenges, secretion of IL-6 and IL-8 in cell culture supernatant were quantified (Fig. 4), as well as the protein expression levels of ABCF1 (Fig. 5) and A20 (Fig. 6). Of the five challenges that were tested, TNF-α and Poly(I:C) increased the protein expression of A20 without changing the expression of ABCF1, and strongly induced the secretion of IL-6 and IL-8. LPS, PGN-SA, and IL-17 failed to induce robust IL-6 and IL-8 secretion (Fig. 4), did not impact ABCF1 expression (Supplementary Fig. 1), and showed a mild induction of A20 (Supplementary Fig. 2). Our data validated the selection of TNF-α and Poly(I:C) for downstream analysis of ABCF1 under challenge conditions.

### ABCF1 does not regulate A20, NF-${\varvec{\kappa}}$ B and IRF-3 mediated inflammatory responses under Poly(I:C) challenge

**Figure 7:**
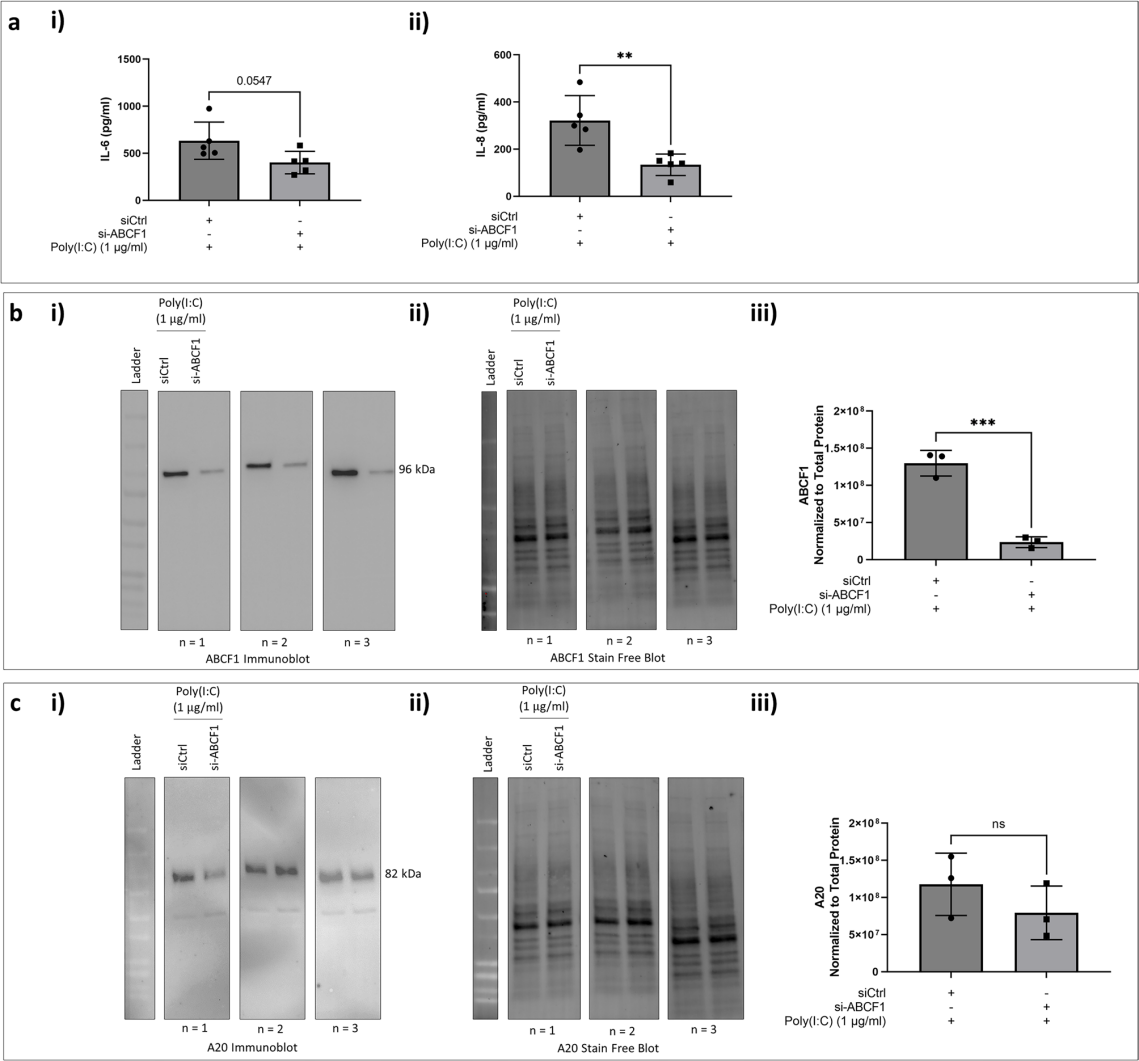
Poly(I:C) induced pro-inflammatory responses on ABCF1 silenced HBEC-6KT in vitro. (**a**) Poly(I:C) (1 μg/ml) induced IL-6 and IL-8 protein production for HBEC-6KT cells with si-ABCF1 treatment. (**b**) Immunoblot with quantification on ABCF1 and (**C**) A20 protein expression in HBEC-6KT cells under Poly(I:C) (1 μg/ml) challenge with and without si-ABCF1 treatment. ELISA data n = 5, immunoblot data n = 3; * *p* ≤ 0.05.

Leveraging our characterization of Poly(I:C) responses in HAECs, we explored the role of ABCF1 with siRNA. ABCF1 knockdown of 81.9% was confirmed in control vehicle and Poly(I:C) stimulated HAECs (Fig. 7b) and was associated with a significant reduction in IL-8 and a trend for reduced IL-6 (Fig. 7a). Under these conditions, we observed no changes in A20 protein expression levels (Fig. 7c).

**Figure 8:**
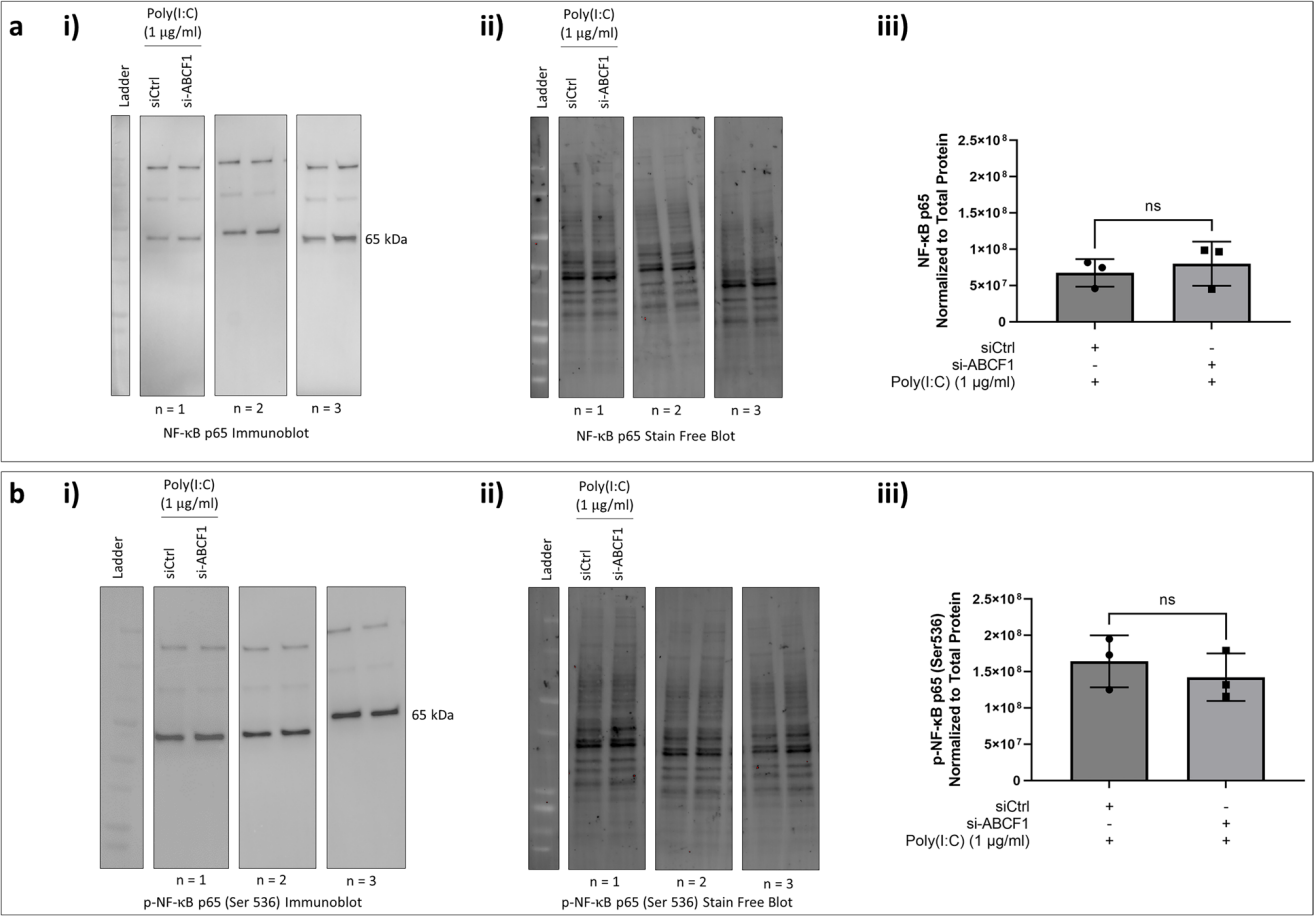
ABCF1 silencing on NF-κB p65 protein expression and activation under Poly(I:C) challenge in HBEC-6KT in vitro. (**a**) Immunoblot with quantification of total NF-κB p65, and (**b**) phosphorylated NF-κB p65 (Ser 536) protein expression in HBEC-6KT cells under Poly(I:C) (1 μg/ml) challenge with and without si-ABCF1 treatment. All studies n = 3; * *p* ≤ 0.05.

Since the observed attenuation of IL-8 is not due to changes in A20 protein expression, we investigated whether the silencing of ABCF1 is affecting the protein expression and activation status of the transcription factors, NF-κB and IRF-3. We observed no changes in total NF-κB protein expression levels (Fig. 8a). Its activation status based on its phosphorylation, also remained unchanged when ABCF1 is silenced under Poly(I:C) challenge (Fig. 8b).

**Figure 9:**
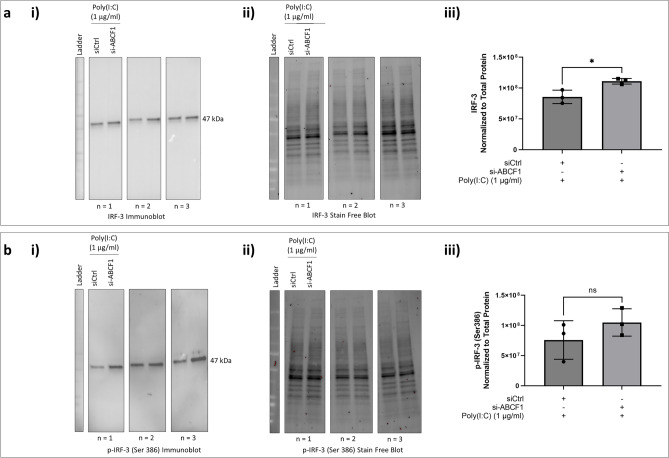
ABCF1 silencing on IRF-3 protein expression and activation under Poly(I:C) challenge in HBEC-6KT in vitro. (**a**) Immunoblot with quantification of total IRF-3, and (**b**) phosphorylated IRF-3 (Ser 386) protein expression in HBEC-6KT cells under Poly(I:C) (1 μg/ml) challenge with and without si-ABCF1 treatment.

Poly(I:C) may signal through IRF-3 and contribute to IL-8 production independent of NF-κB signaling. We therefore analyzed IRF-3 and its phosphorylation. We observed an increase in total IRF-3 protein expression levels (Fig. 9a), while no changes in level of phosphorylation were observed (Fig. 9b).

Collectively, these studies demonstrate that Poly(I:C) induced IL-8 is regulated by ABCF1 via pathways independent of NF-κB and IRF-3 activation.

### ABCF1 does not regulate A20 and NF-κB mediated inflammatory responses under TNF-α challenge

**Figure 10:**
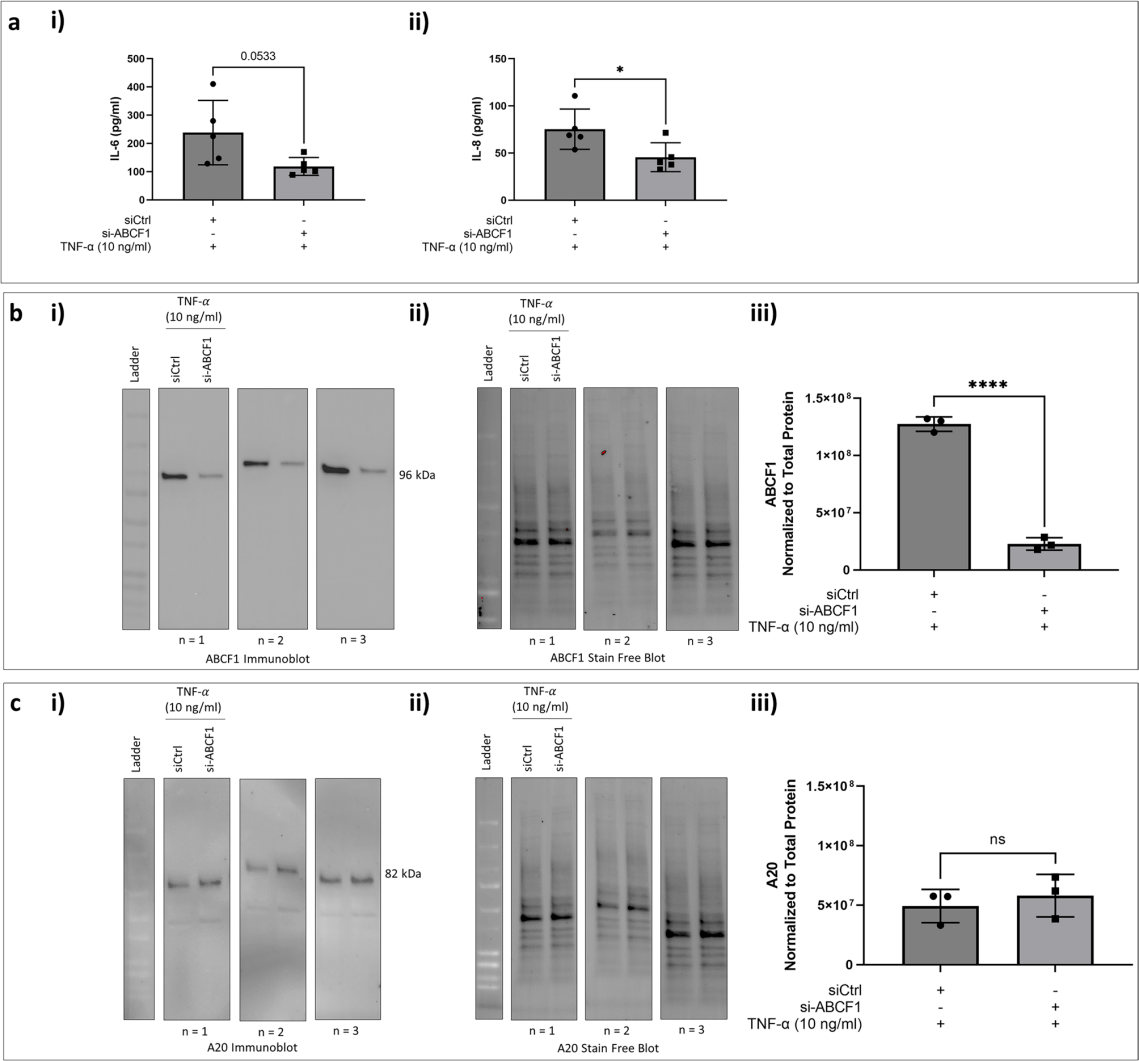
TNF-α induced pro-inflammatory responses on ABCF1 silenced HBEC-6KT in vitro. (**a**) TNF-α (10 ng/ml) induced IL-6 and IL-8 protein production for HBEC-6KT cells with si-ABCF1 treatment. (**b**) Immunoblot with quantification on ABCF1 and (**c**) A20 protein expression in HBEC-6KT cells under TNF-α (10 ng/ml) challenge with and without si-ABCF1 treatment. ELISA data n = 5, immunoblot data n = 3; * *p* ≤ 0.05.

It remained possible that ABCF1 regulates NF-κB signaling in a context dependent manner that was influenced by the stimulus. We therefore investigated whether ABCF1 regulates TNF-α mediated pro-inflammatory responses in HAEC using identical approaches to our Poly(I:C) experiments. Consistent with Poly(I:C) data, we observed a significant reduction in IL-8 levels and a trend for reduced IL-6 (Fig. 10a) with ABCF1 knockdown of 82.2% (Fig. 10b). Again, no changes in A20 protein expression levels were observed with ABCF1 silencing (Fig. 10c) under TNF-α stimulation conditions we have shown to induce A20 (Fig. 6).

**Figure 11:**
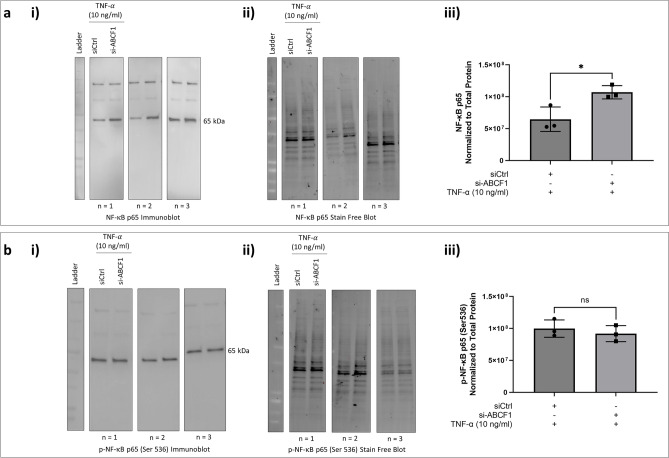
ABCF1 silencing on NF-${\upkappa }$ B p65 protein expression and activation under TNF-α challenge in HBEC-6KT in vitro. (**a**) Immunoblot with quantification of total NF-κB p65, and **c)** phosphorylated NF-κB p65 (Ser 536) protein expression in HBEC-6KT cells under TNF-α (10 ng/ml) challenge with and without si-ABCF1 treatment. All studies n = 3; * *p* ≤ 0.05.

We next investigated whether silencing of ABCF1 expression under TNF-α stimulation has any impact on NF-κB expression and activity. We observed an increase expression level of total NF-κB with ABCF1 silencing under TNF-α stimulation (Fig. 11a) although the phosphorylation status of NF-κB, was not changed under these conditions (Fig. 11b).

Collectively, these studies demonstrate that TNF-α induced IL-8 is regulated by ABCF1 via pathways independent of NF-κB activation.

## Discussion

Explorations into the role ABCF1 has in modulating respiratory mucosal immune responses are grounded in reports that this protein has a diverse range of activities including translation initiation, viral sensing in the cytosol, and polarization of immune cell phenotype^9, 12, 13^. Our group previously confirmed the expression of ABCF1 at the gene and protein level in the airway epithelium. Using siRNA-mediated knockdown on ABCF1 followed by a viral cytosolic nucleic acid challenge in airway epithelial cells, our findings suggested that ABCF1 was not involved in regulating antiviral responses but instead mediates an immune response involving TLR-3/4 signalling pathways. One of the genes that was significantly differentially expressed in these signalling pathways under ABCF1 silencing and viral challenge included *TNFAIP3*, expressed as A20 protein, a ubiquitin ligase and deubiquitinating enzyme that supresses the NF-κB transcription factor activation^15^. The present manuscript explored the regulatory and functional relationship between ABCF1 and A20 in HAECs under basal unstimulated and pro-inflammatory challenge conditions.

For this study, we investigated the function of ABCF1 in an immortalized human bronchial epithelial cell line called HBEC-6KT. This cell line was developed using retroviral expression of Cdk4 and hTERT to promote cell proliferation and extend their lifespan. This was developed in cells that were obtained from bronchial biopsies from areas of the lungs that were histologically not involved with cancer. Unlike other cell lines that are immortalized by viral oncogenes, the Cdk4/hTERT cells are phenotypically normal, and they do not exhibit malignant characteristics. Karyotyping analyses have been conducted in similar HBEC lines and demonstrated that the approach of over-expressing Cdk4/hTERT results in cells that are genetically more like parental cells than HPV-immortalized cell lines and distinct from lung cancerous cell lines such as BEAS-2B^18^. It remains possible that the HBEC-6KT cell line used has interruptions in genomic regions that regulate ABCF1 expression and could lead to haploinsufficiency. Routine karyotyping of cell lines has been suggested in the past^29^. The rationale for choosing HBEC-6KT was to ensure continuity with our earlier research that has demonstrated that the expression of ABCF1 protein is conserved in both primary human airway epithelial cells and HBEC-6KT HAECs with functional links to innate immune responses^14^.

As no selective pharmacological inhibitor for ABCF1 has been reported and evidence that homozygous deletion of ABCF1 is fatal in mice, we have pursued ABCF1 siRNA approaches in this paper and past reports^14^. Previously, our group showed that with 35% ABCF1 silencing, there was no observed changes in HAEC secreted IL-8 under Poly(I:C) challenge in vitro^14^. In contrast to our previous findings, increased efficiency in gene silencing that produces approximately 80% ABCF1 knockdown led to a decreasing trend in Poly(I:C) induced secretion of IL-8 when compared to silencing control. Compared to our gene knockdown efficiency, Hsu et al*.* demonstrated that with 50–70% knockdown of ORMDL3 gene expression using siRNA in A549 and 1HAE airway epithelial cell lines, they observed no changes to the levels of NF-κB-induced IL-6 and IL-8 under TNF-α and LPS challenges^30^. However, in our study we were able to achieve sufficient knockdown of ABCF1 to observe changes in the secretion of these cytokines. This was done by ensuring our cell lines were treated with siRNA at an appropriate confluency of 70% and at a concentration of 25 nM for no more than 24 h to avoid cell death, potentially due to off target effects that accompany this approach^31^. For the purpose of investigating our previous findings, we carried forward with the use of subconfluent HAECs in this study, maintaining continuity with our earlier research. However, future approaches with conditional deletion of ABCF1 in human cell lines using CRISPR-Cas9 KO or novel selective inhibitors of ABCF1 and the use of cultures grown at air liquid interface (ALI) are warranted to further consolidate a role for this protein in regulating immune responses^32–34^.

The expression and function of A20 in HAECs has been defined in the context of pro-inflammatory responses and long-acting β_2_-adrenoreceptor agonists (LABAs)/glucocorticoid combination treatments^24, 35^. While characterizing the differences between the pro-inflammatory responses in HAECs and alveolar macrophages, it has been proposed that the response to TLR-3 and TLR-4 agonists differs between these two cell types. They first demonstrated that under basal conditions, gene expression of A20 was unchanged in both HAECs and alveolar macrophages. Under Poly(I:C) stimulation, they showed that compared to alveolar macrophages, HAECs had a stronger pro-inflammatory response and higher A20 protein expression. While under LPS stimulation, alveolar macrophages had a stronger pro-inflammatory response but A20 levels was not impacted^24^. A separate group proposed that combination treatment in HAECs can augment A20 protein expression to repress NF-κB mediated pro-inflammatory responses. Using HAECs, they demonstrated that LABAs augments A20 expression to negatively regulate NF-κB and consequently improve the anti-inflammatory properties by glucocorticoids^35^. Our data confirm the protein expression of A20 in HAECs under basal conditions and demonstrate that ABCF1 silencing by 80% fails to change A20 protein expression levels. This contrasts to previous research where a group observed a reduced expression of A20 in *macrophages* with ABCF1 silencing under basal conditions^13^. The contrasting results may owe to the cell type selected, as HAECs and macrophages have shown different A20 expression levels and therefore may be regulated by different mechanisms^24^. The regulation of A20 is intertwined with the transcription factors NF-κB and IRF-3, through negative feedback loops that enable shutting off pro-inflammatory signaling by A20^36^. As ABCF1 silencing failed to regulate A20 expression under basal conditions, we sought to deeper characterize this pathway and define NF-κB and IRF-3 expression and activation. With an immunoblot on total NF-κB p65, a subunit of NF-κB, and its activated phosphorylated form, we showed that the knockdown of ABCF1 led to no changes to the protein expression of total NF-κB p65, and its activation when compared to the silencing control under basal unstimulated conditions. The protein expression of total IRF-3 and its activated phosphorylated form was also unchanged. Collectively, our data do not demonstrate any relationship between ABCF1 and A20 expression and NF-κB and IRF-3 activated signalling pathways in HAECs under basal unstimulated conditions.

It is possible that baseline A20 expression levels are not regulated by ABCF1, while dynamic upregulation of A20 may be dependent on ABCF1, owing to the latter protein’s proposed roles in translation initiation and modulation of immune responses^9, 13^. We therefore investigated a variety of pro-inflammatory challenges that are known to be regulated by A20, including TNFR, TLR-2, TLR-3, TLR-4, and IL-17R induced signalling pathways^15, 25, 26, 37^. Under viral stimulation, downstream of TLR-3 signalling, A20 directly interacts with IRF-3 kinases, NF-κB-activating kinase/Traf family member-associated NF-κB activator-binding kinase 1 (NAK/TBK1) and IKK-ι/IKK-ε to inhibit IRF-3 phosphorylation, dimerization, translocation to the nucleus and downstream expression of interferon stimulation response element (ISRE) transcripts^16, 38^. Consistent with literature, TNF-α and Poly(I:C) were able to induce IL-6 and IL-8 cytokine secretion and an associated upregulation in A20 expression in a concentration-dependent manner in HAECs^24, 27, 28, 39^. In contrast, those stimuli (LPS, IL-17, PGN-SA) that showed more modest induction of IL-6 and IL-8 failed to induce A20 expression. Prior research showed that ABCF1 silencing in macrophages led to a reduction in IRF-3 dimerization and phosphorylation with and without LPS stimulation^13^. In our HAEC model, in vitro LPS stimulation was unable to induce a strong pro-inflammatory response which is consistent with previous reports with primary HAECs^24^. The data suggest that there may be cell specific responses to LPS that lead to A20 regulation. Taken together, our data describe a relationship where stimuli that induce IL-6 and IL-8 are accompanied by a concomitant expression of A20, a negative regulator of pro-inflammatory responses. These experimental conditions enable mechanistic studies aimed at defining a role for ABCF1 in regulating A20 expression and function.

Since no changes were observed for protein expression levels of A20 under TNF-α and Poly(I:C) challenge, we investigated whether there were changes to the expression levels of the transcription factors NF-κB and IRF-3. The protein expression of total NF-κB and its activation was unchanged, however, there was an increased expression of total IRF-3, suggesting that increased activation could be possible. Although, the level of IRF-3 phosphorylation remained unchanged when compared to the control, suggesting that while there was a potential for increase IRF-3 activity, its activation was not affected by ABCF1 silencing under Poly(I:C) challenge.

Downstream of TNFR signalling with TNF-α stimulation, A20 can negatively regulate the activation of NF-κB by inhibiting IκB kinase (IKK) phosphorylation through its protein ubiquitin ligase and deubiquitinatinase activities. This consequently prevents NF-κB from translocating to the nucleus for gene transcription and prevents Protein Kinase A (PKA) from phosphorylating NF-κB subunits, including p65^15, 40^. Phosphorylation of p65, is known to be essential for the stability, degradation, and transcriptional activity of NF-κB with factors involved in gene transcription, including CBP/p300^40^. Early studies characterizing ABCF1 used TNF-α stimulation on fibroblast-like synoviocytes, where an increase in *Abcf1* gene expression was observed and led to the suggestion that it was involved in regulating inflammation^17^. Our study demonstrated that with ABCF1 silencing under TNF-α challenge in vitro, there was a reduction in secreted IL-8 and a more modest trend for reduced IL-6. While there were no changes to the protein expression levels of A20, we observed an increase expression level of total NF-κB. This suggests that there is a potential for increased NF-κB activation and downstream activities. However, the phosphorylation of NF-κB with ABCF1 silencing under TNF-α challenged was unchanged when compared to the silencing control. This finding suggests that while there is an increase potential for NF-κB activation, this was not affected by ABCF1 silencing under the TNF-α challenge.

In conclusion, our study demonstrated the roles of ABCF1 in regulating innate immune responses under pro-inflammatory challenges. We demonstrated that under basal conditions, ABCF1 silencing led to no changes to the protein expressions of A20 and the transcription factors, NF-κB and IRF-3, and its activation. However, we did observed changes in levels of secreted pro-inflammatory cytokines, IL-8 and IL-6, as well as increased protein expression of total NF-κB and IRF-3 under the challenges. These findings suggest ABCF1 may have a role in regulating pro-inflammatory responses in HBEC-6KT HAECs, but its exact function will require further investigation. We propose that ABCF1 protein interactome may consist of interactors that are known to be involved in intracellular signalling pathways to regulate innate immune responses. Our data supports future investigations into the functional role of ABCF1 using CRISPR-Cas9 KO cells with an unbiased protein–protein interaction experiments to gain insights into its function in HAECs.

### Supplementary Information

Supplementary Figures.

## Data Availability

The datasets generated and analysed during the course of the study are available from the corresponding author on reasonable request.
