# Supplementary figures and images for: TNF-α and Poly(I:C) induction of A20 and activation of NF-κB signaling are independent of ABCF1 in human airway epithelial cells
Source: Sci Rep. 2023 Sep 7;13:14745. doi: 10.1038/s41598-023-41990-w (PMC10485056; doi:10.1038/s41598-023-41990-w)

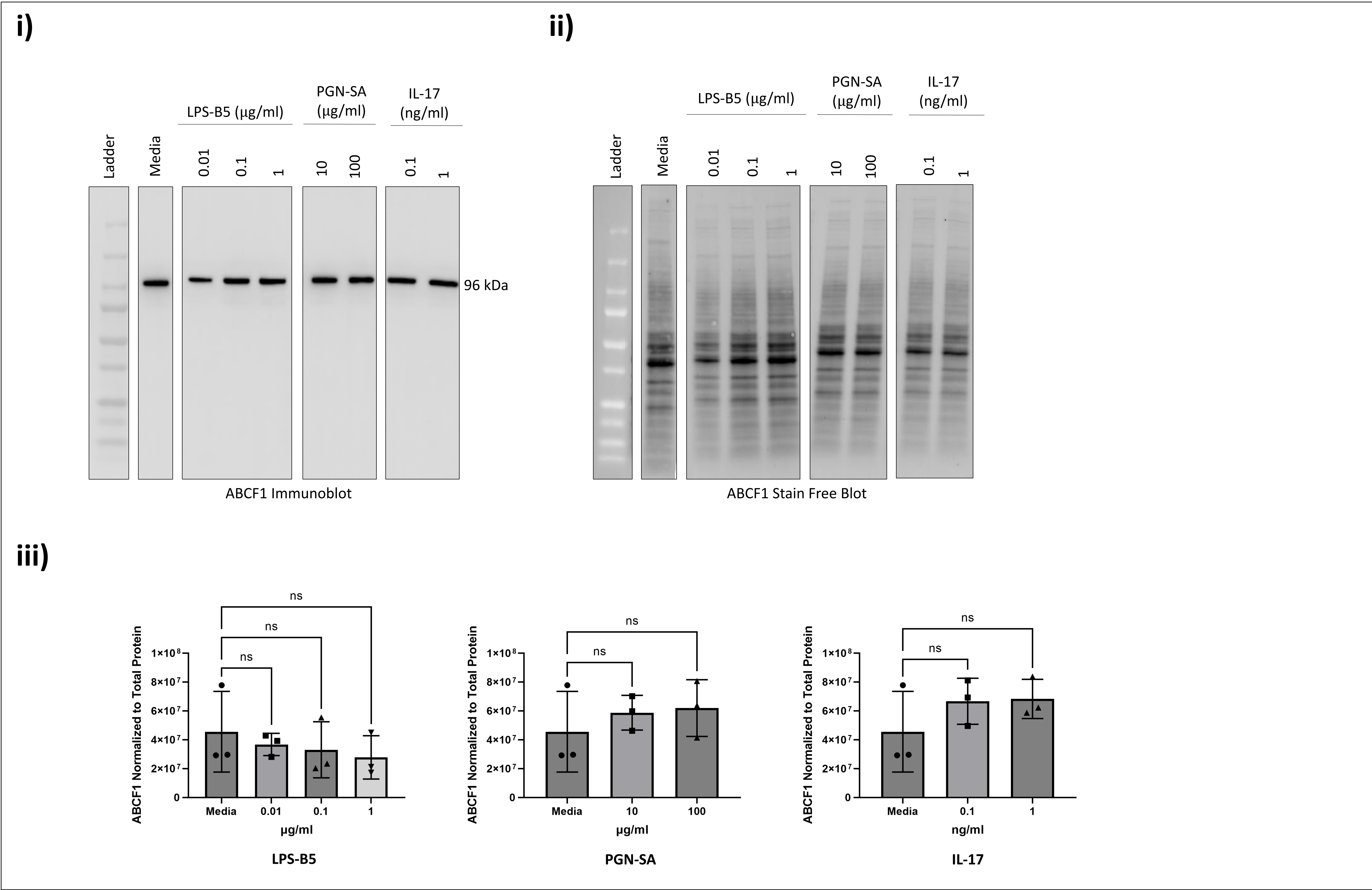

i)

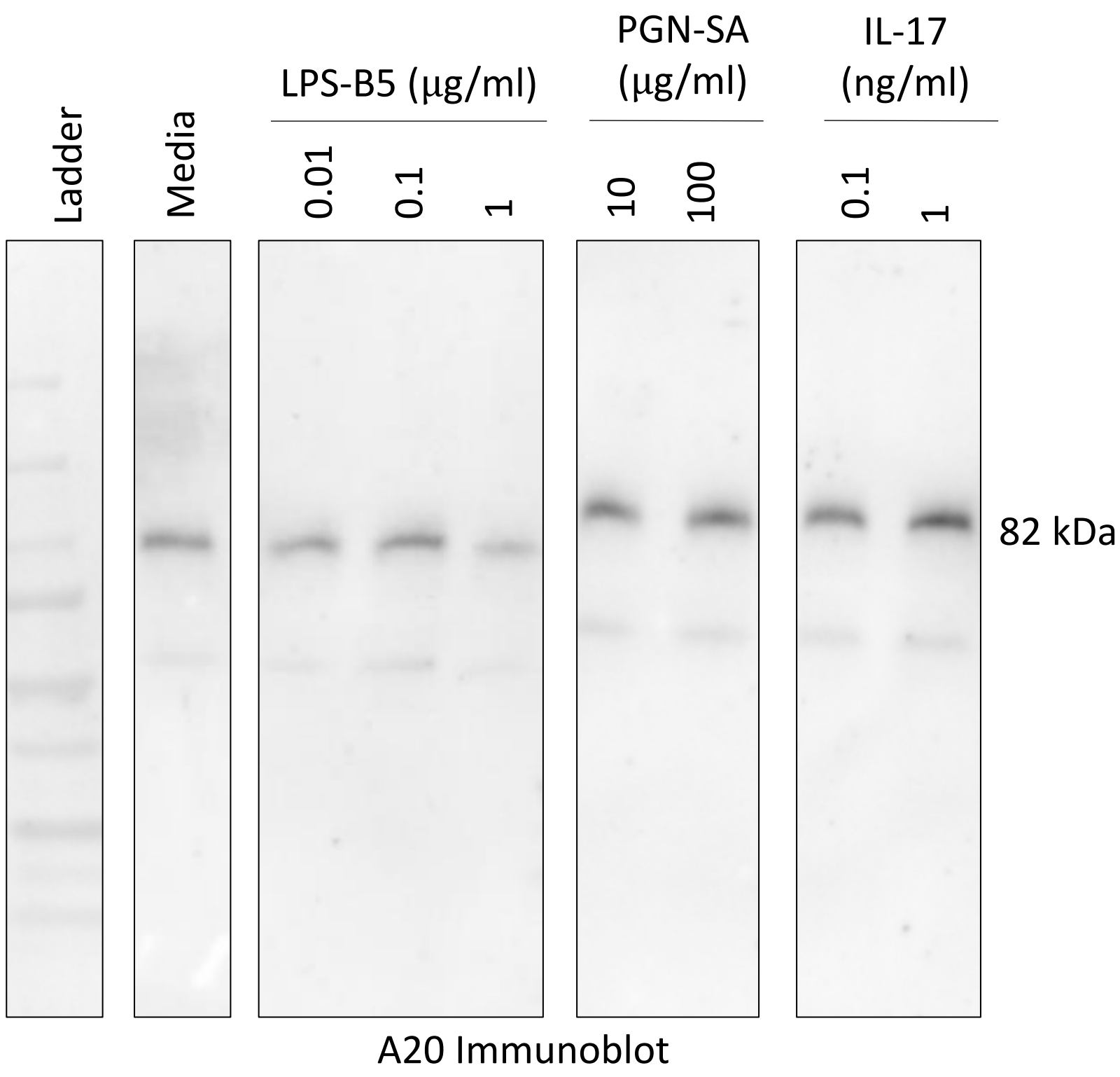

ii)

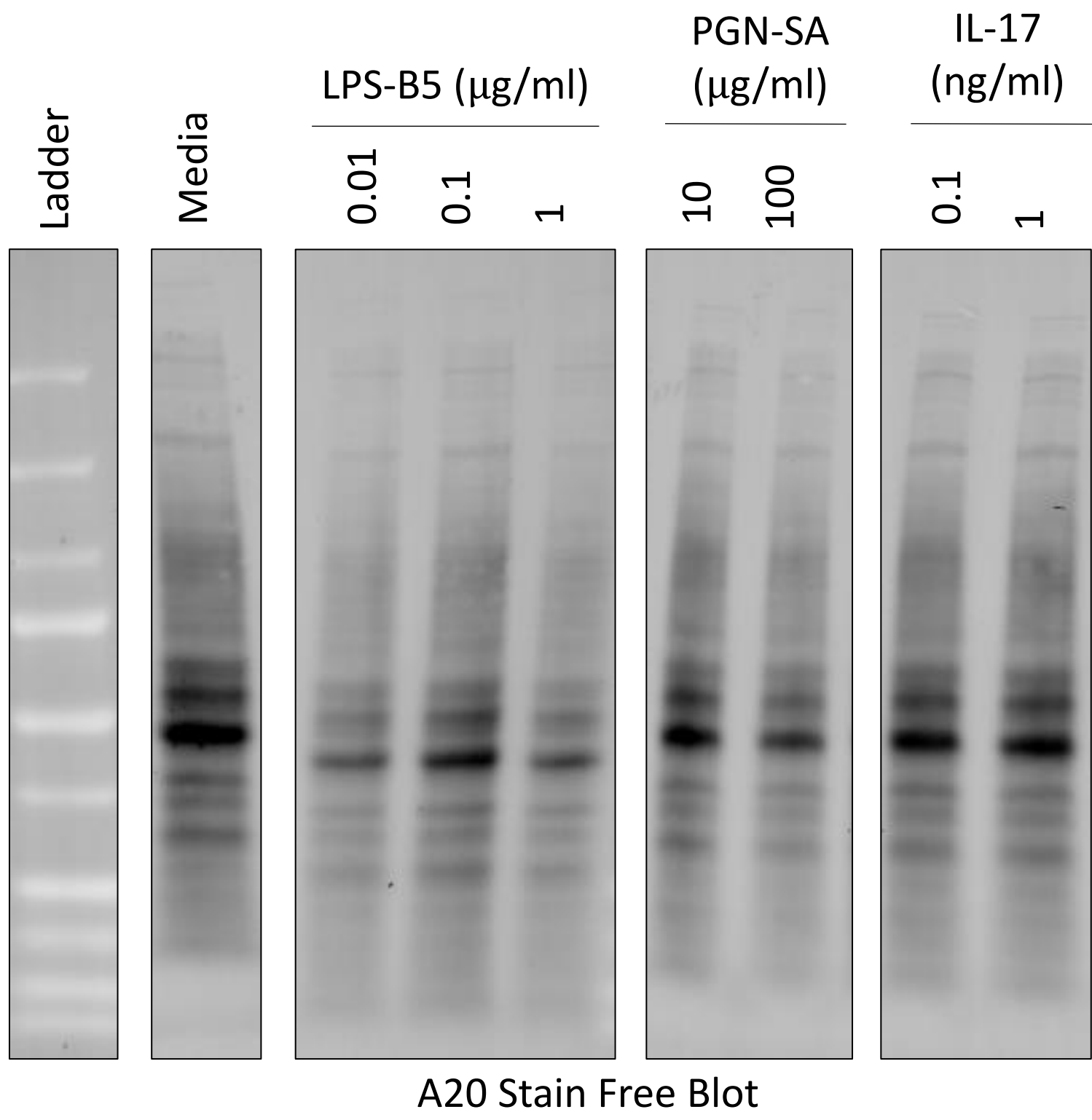

iii)

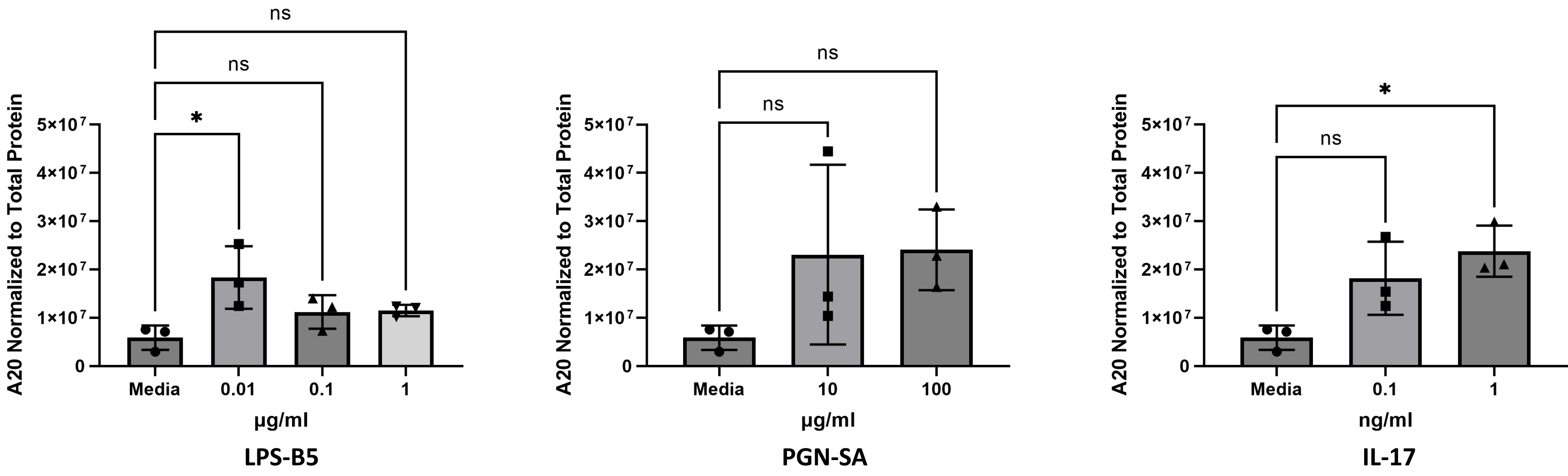

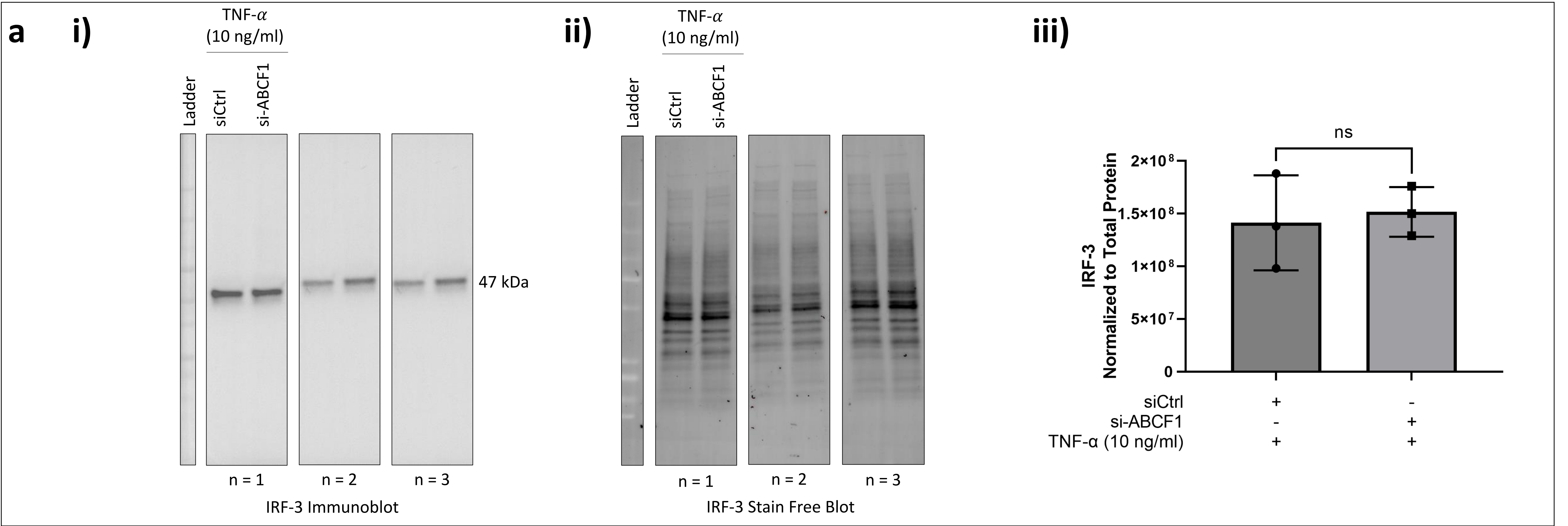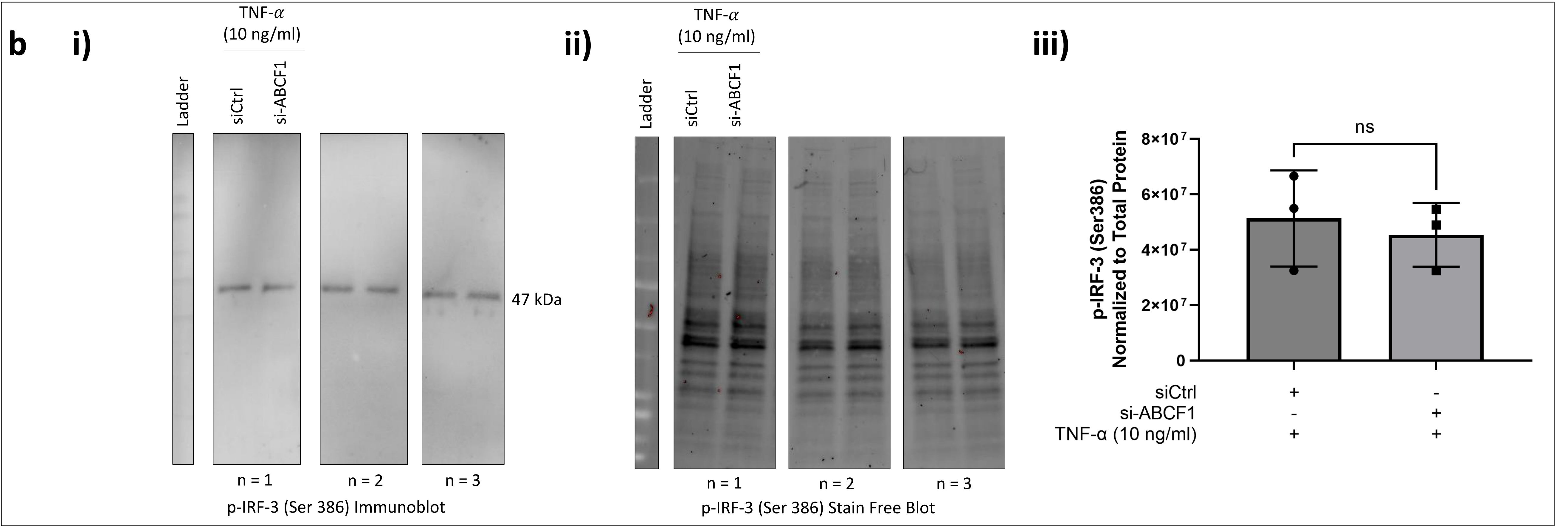

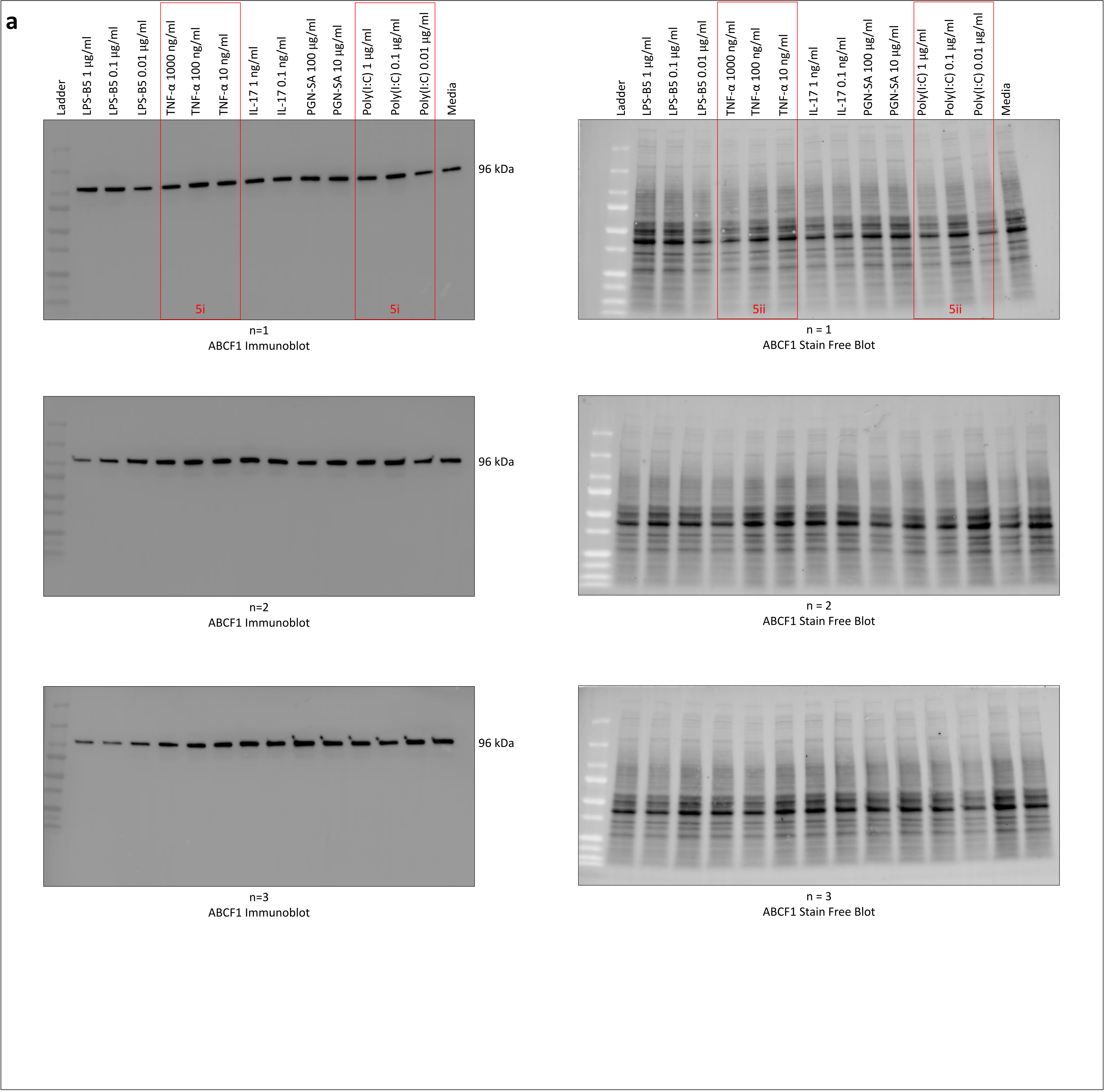

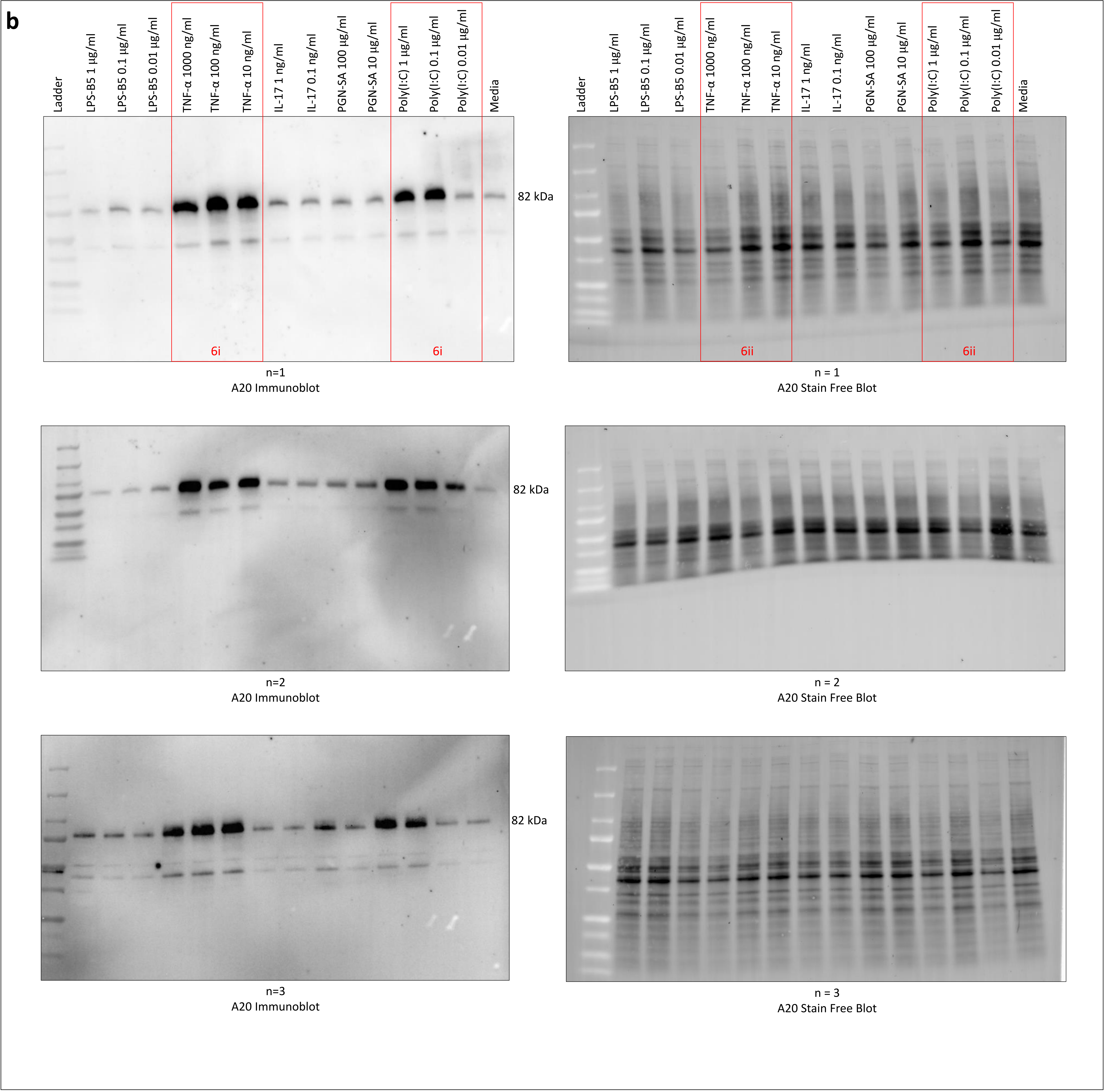

a

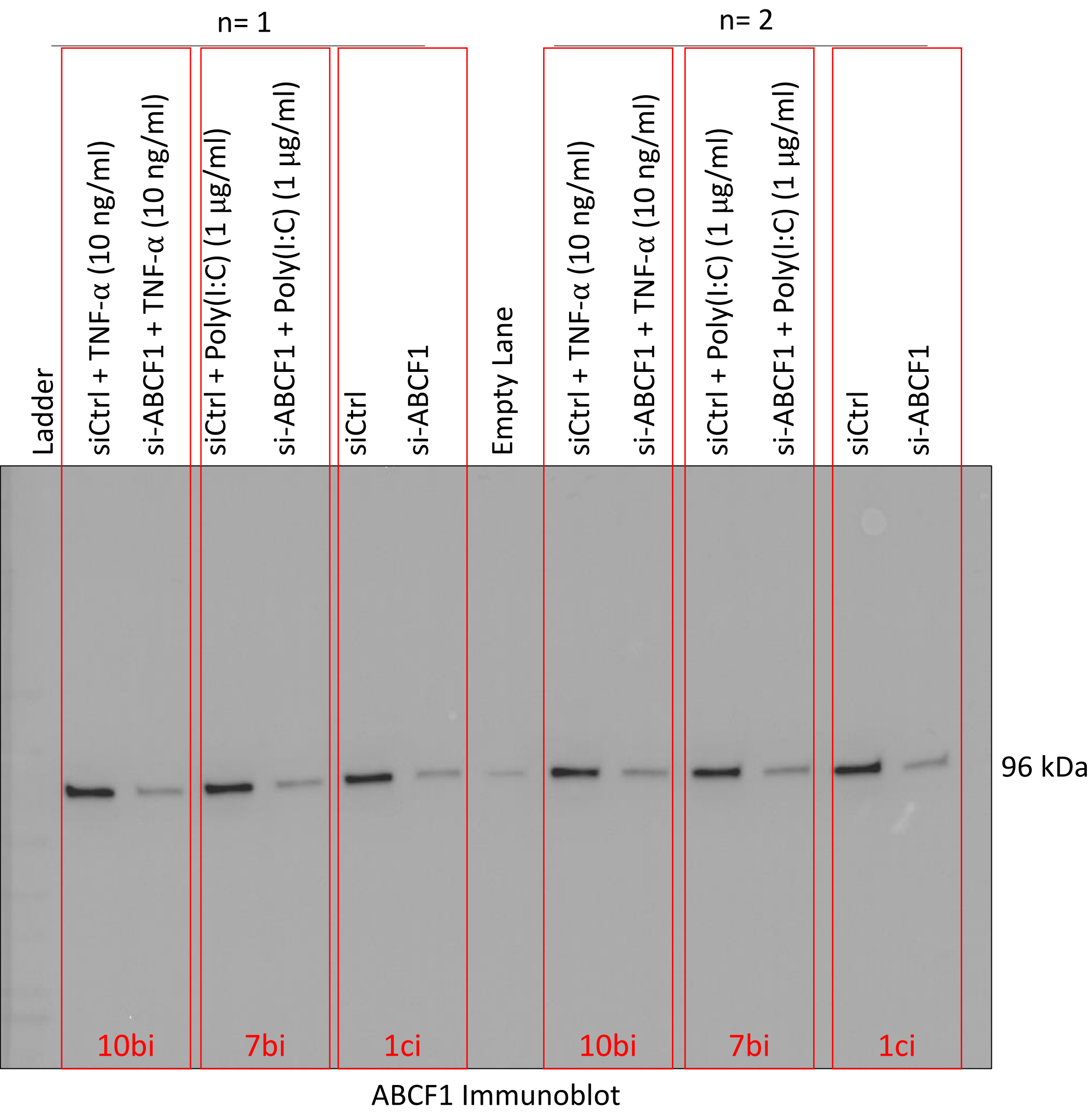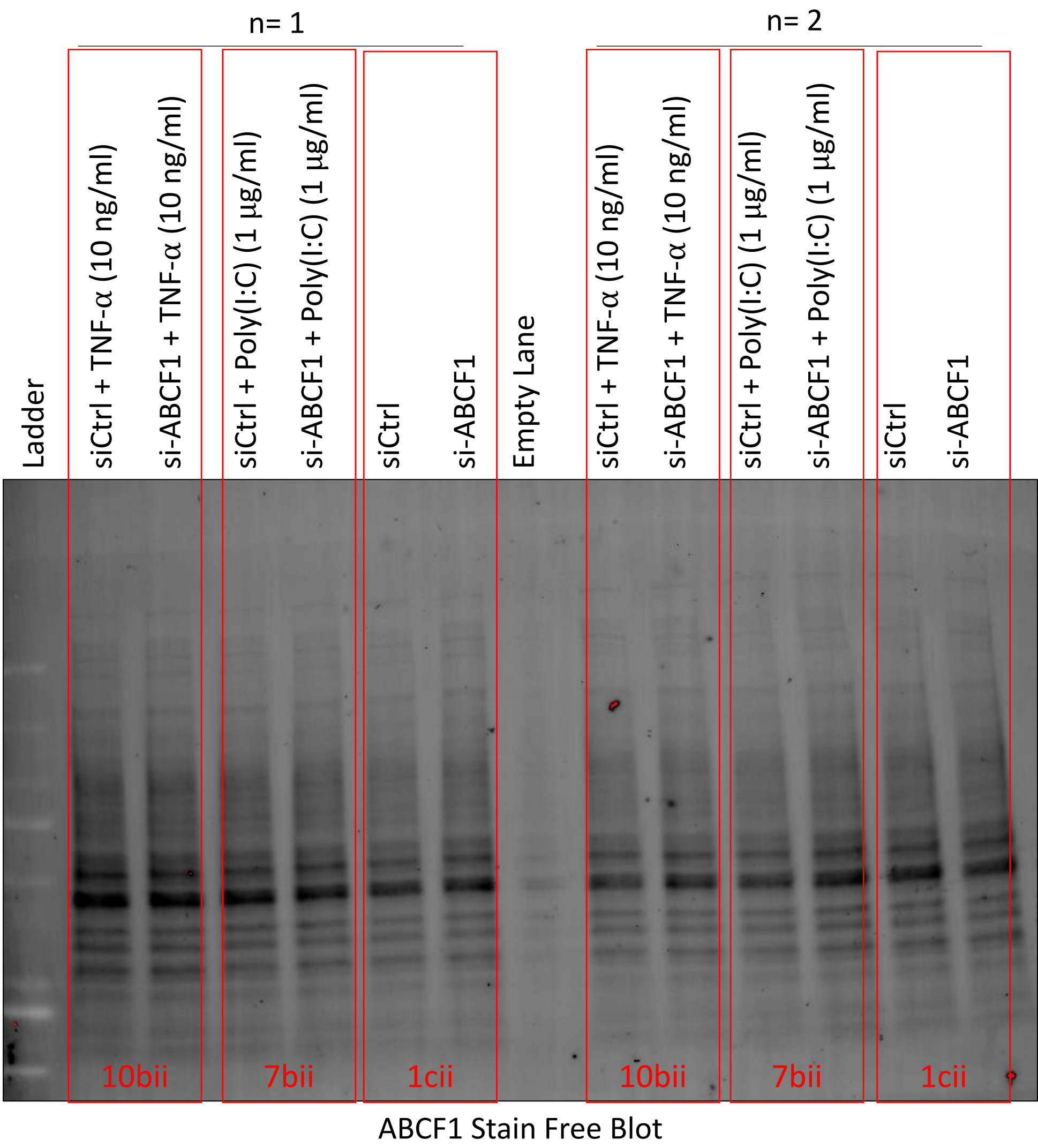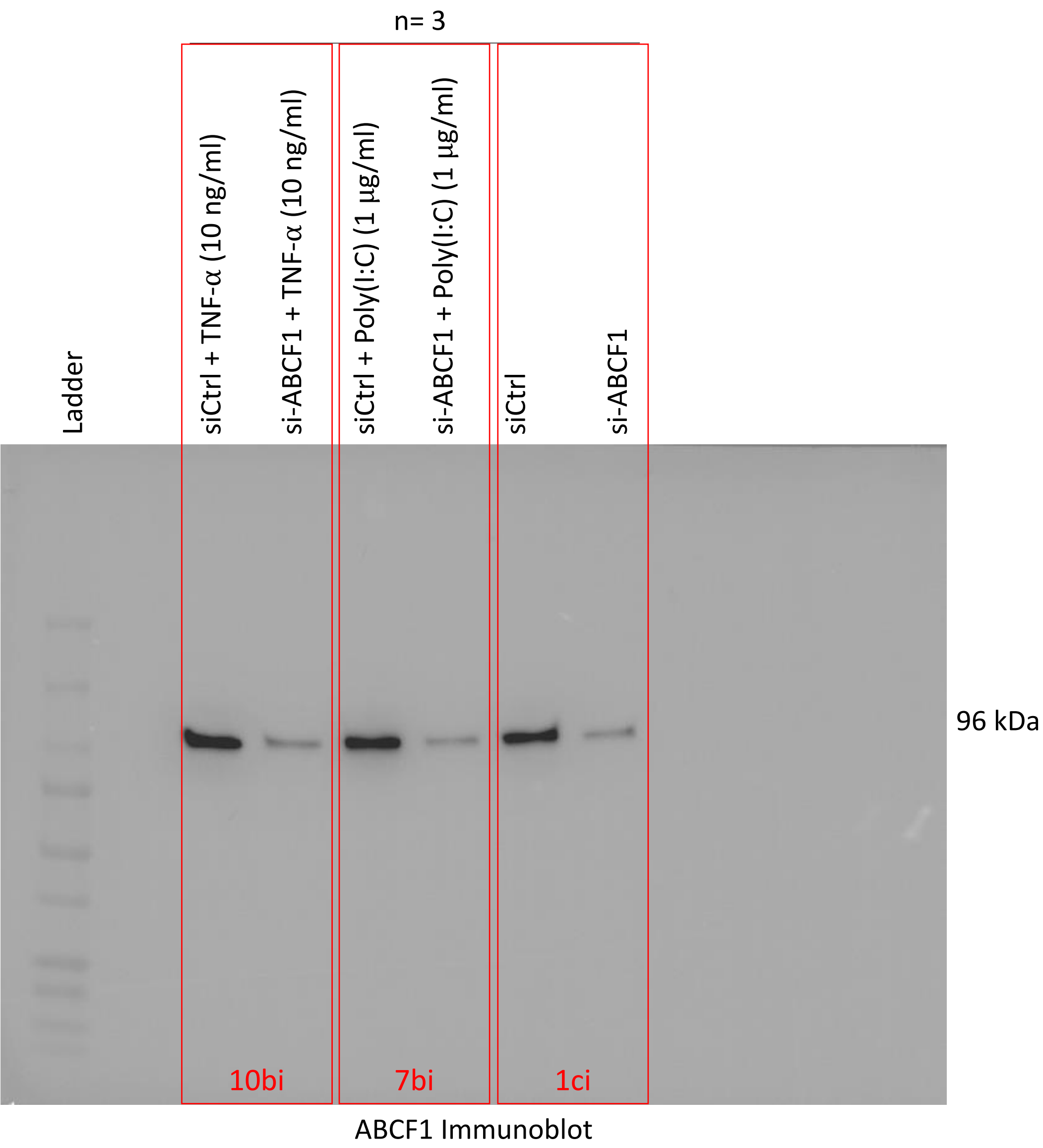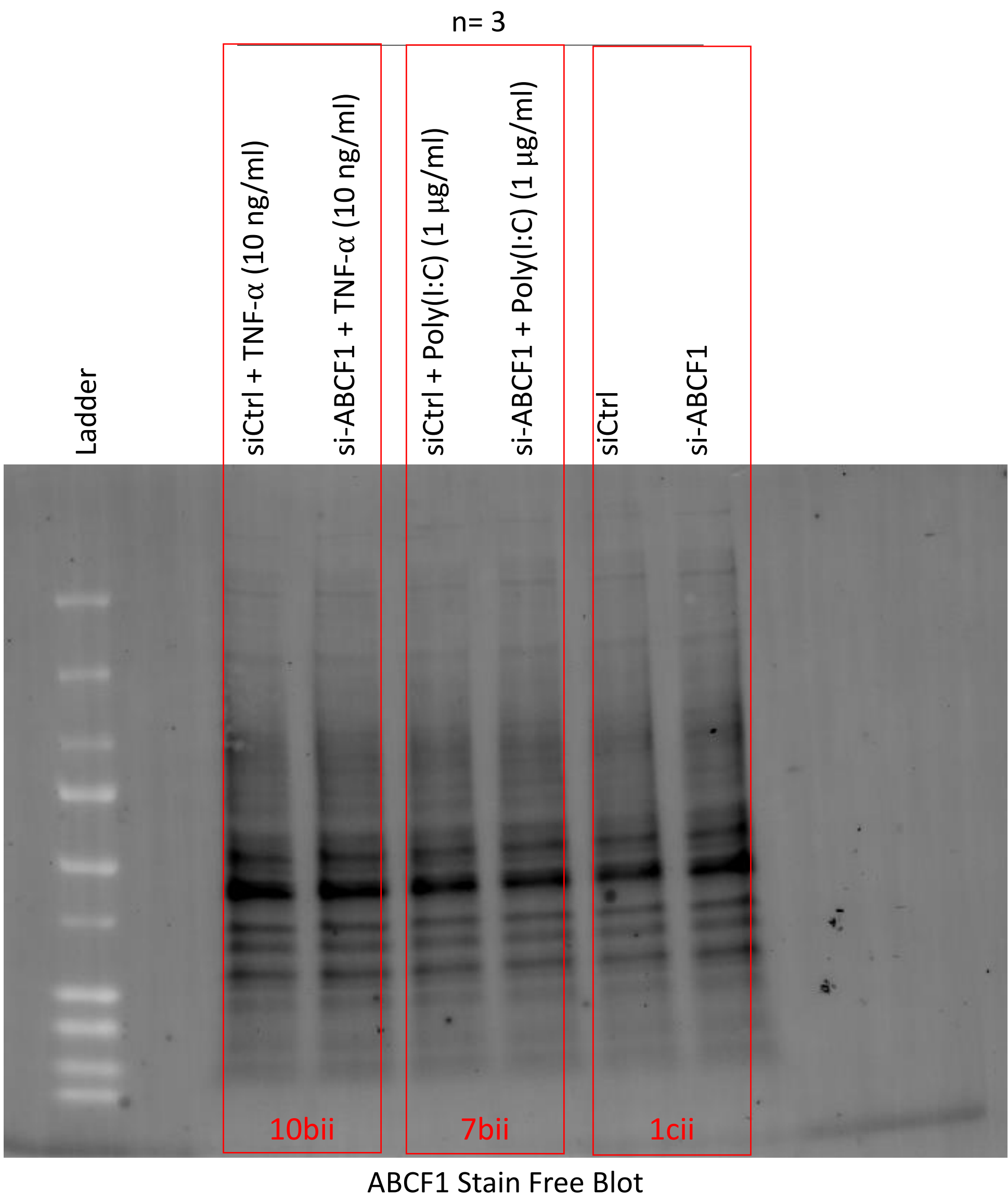

**b**

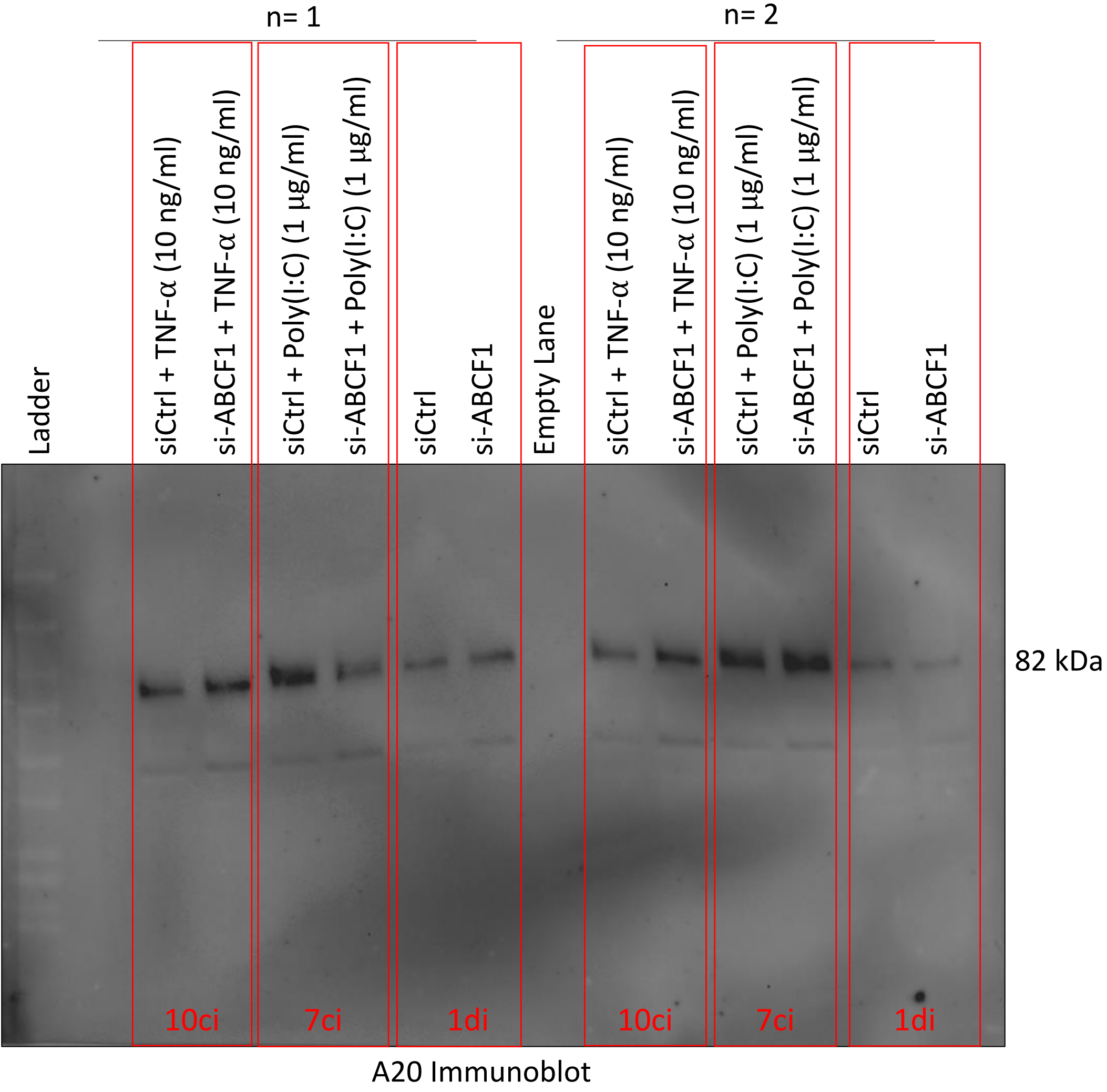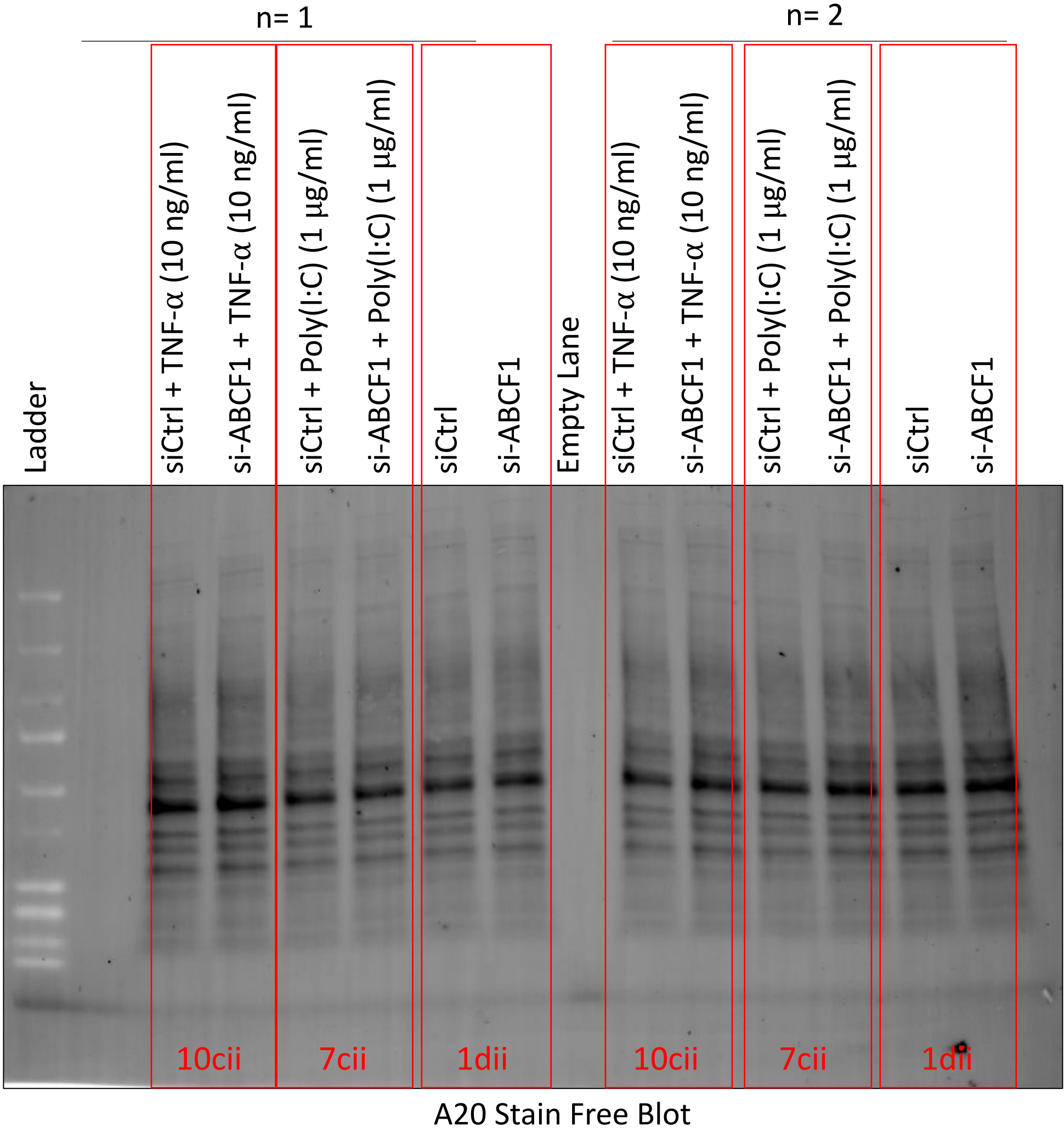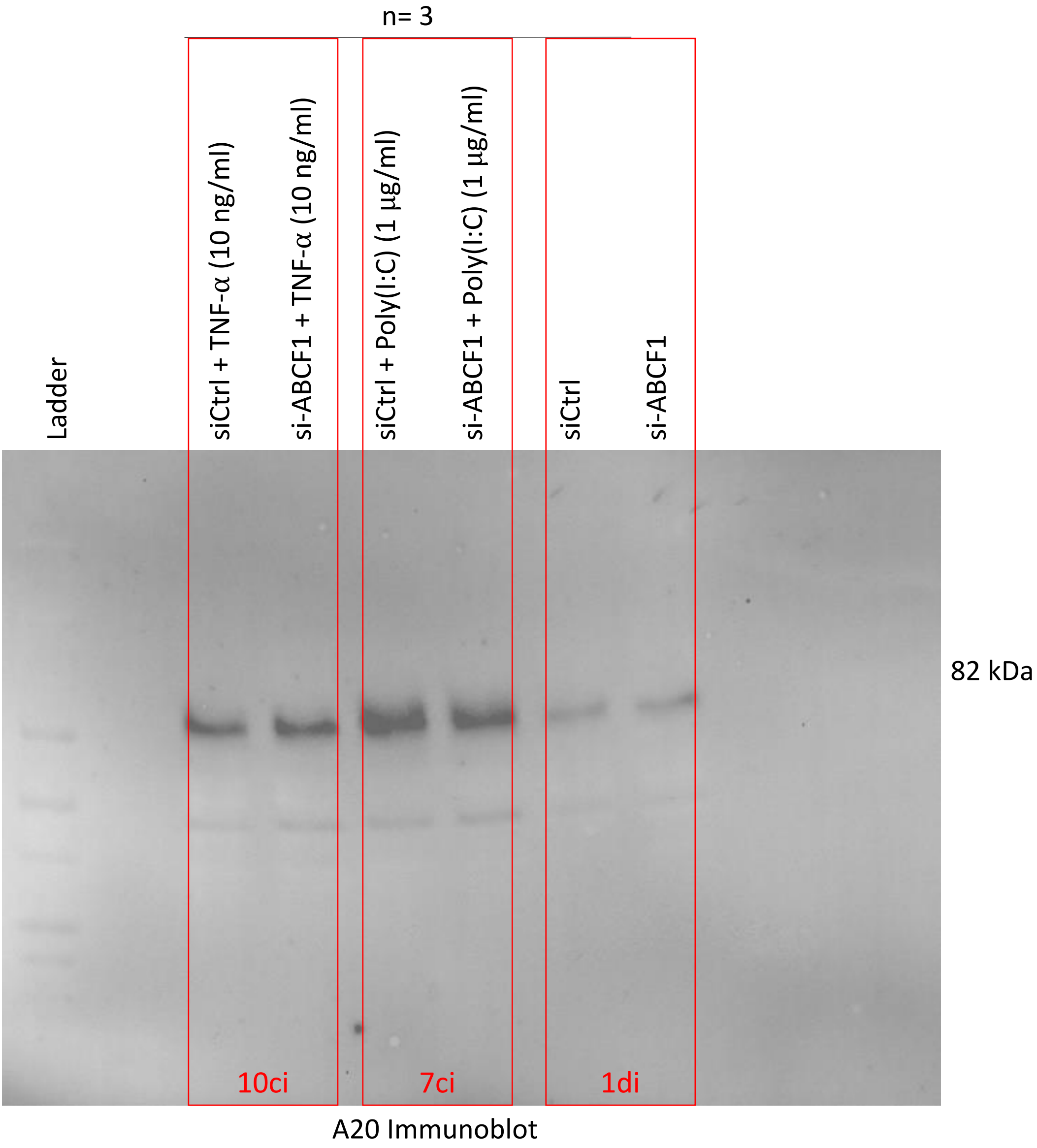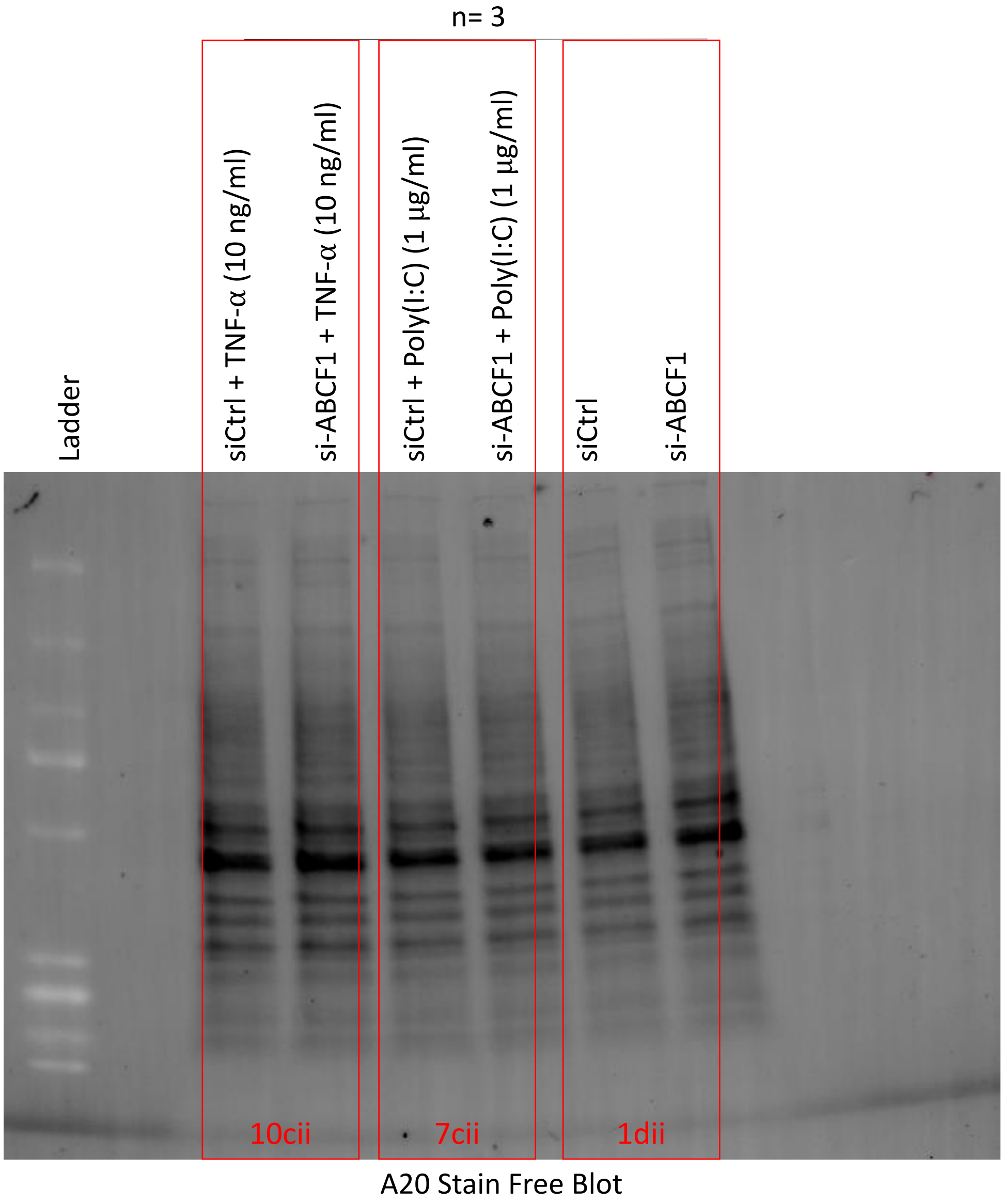

c

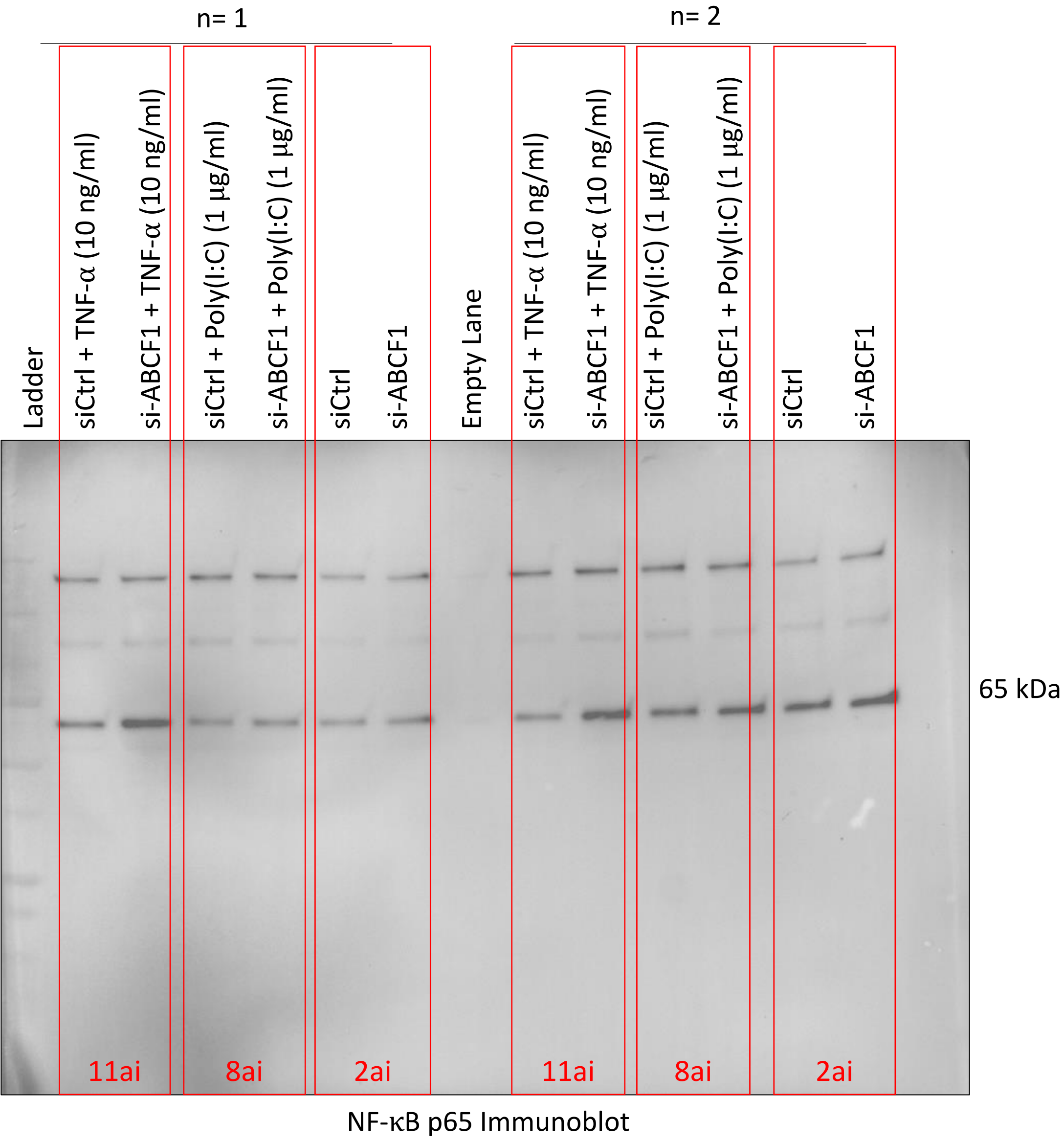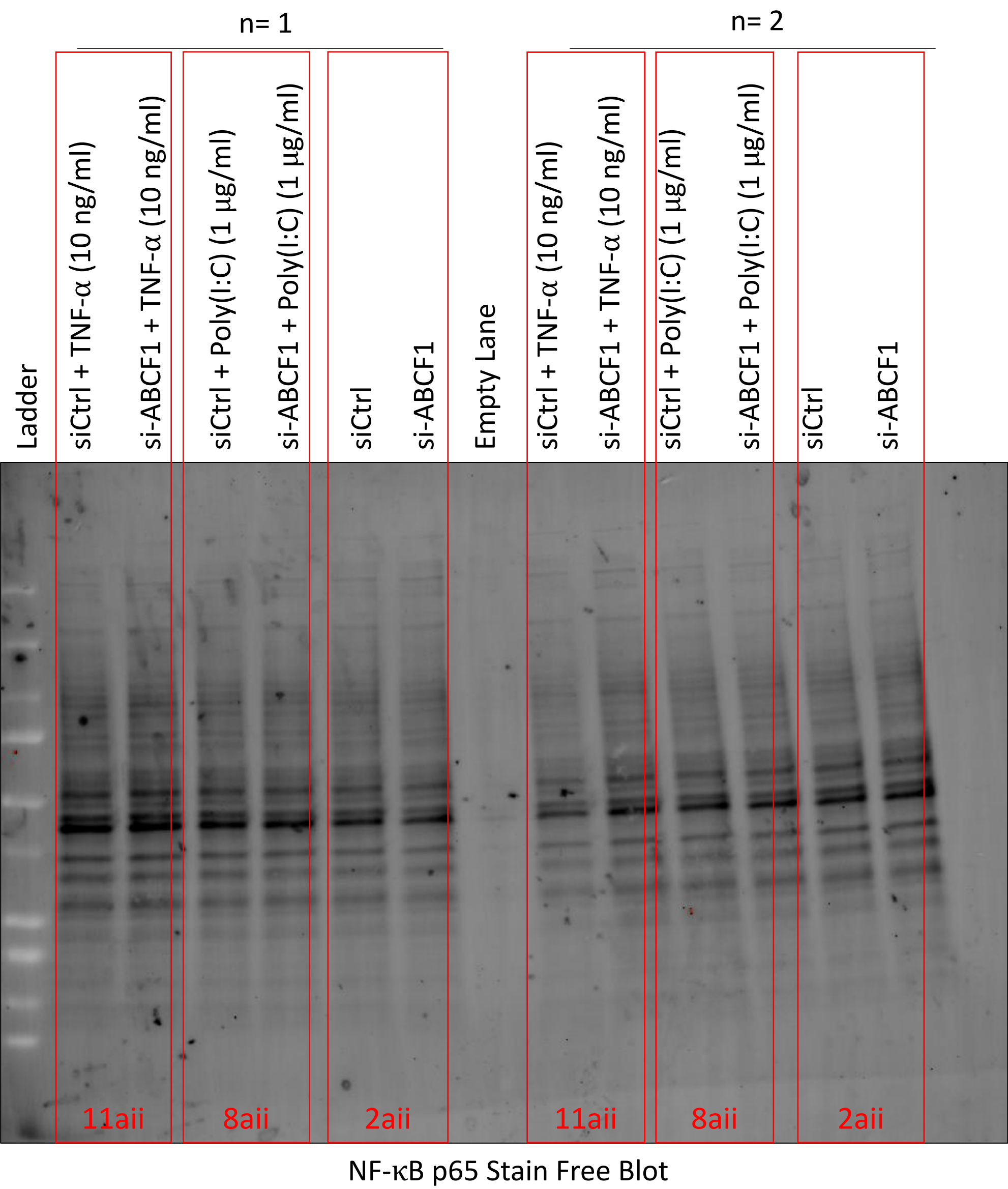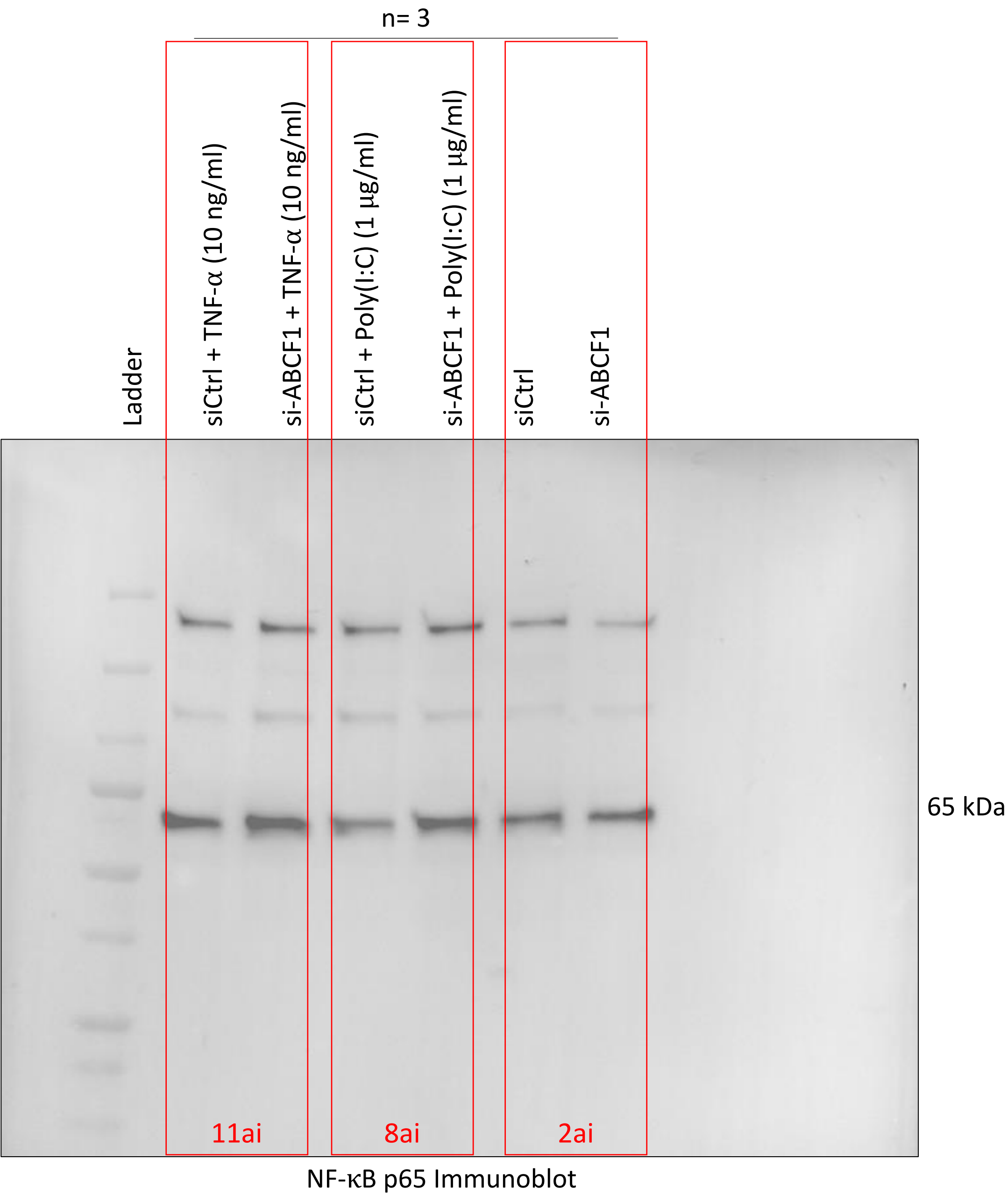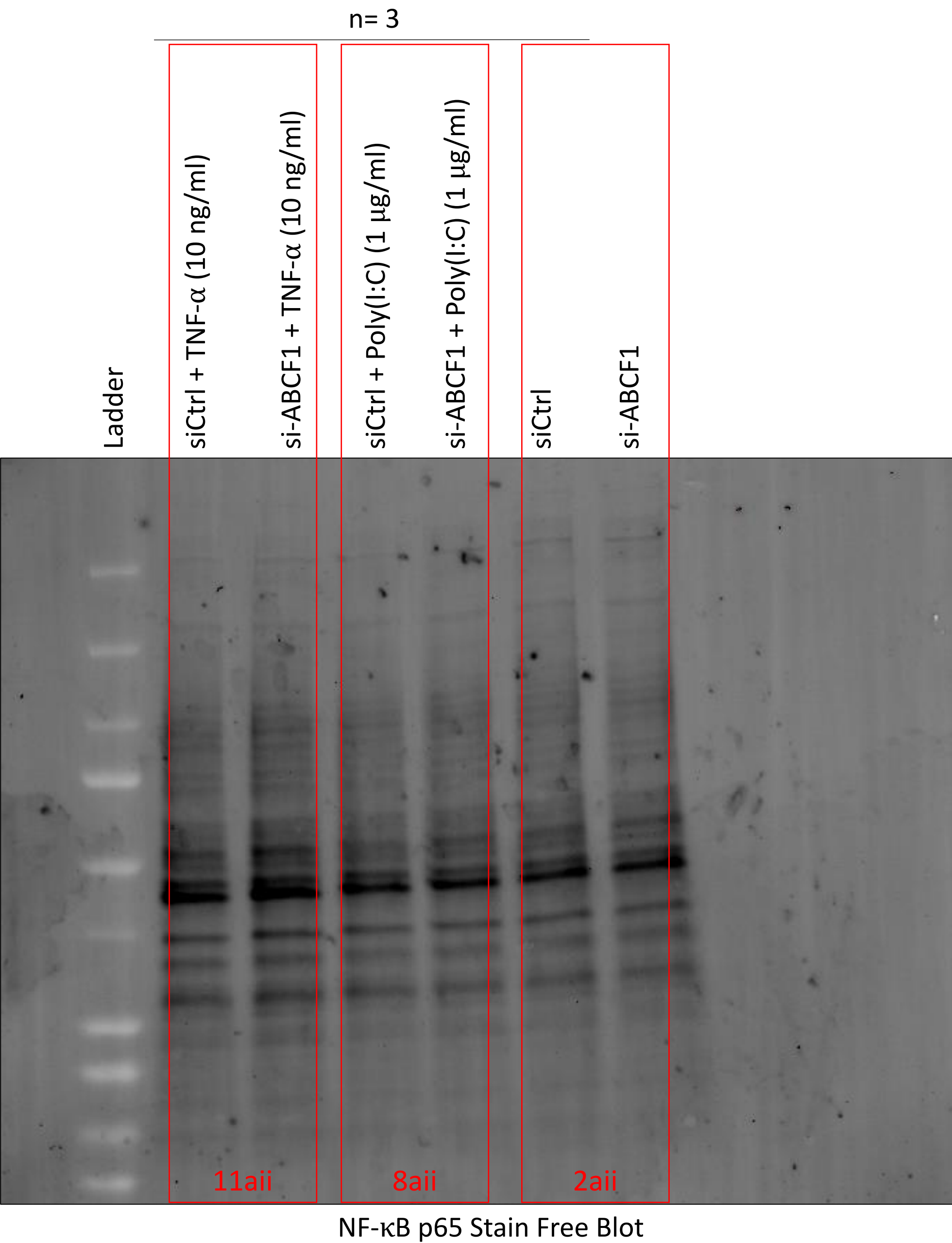

d

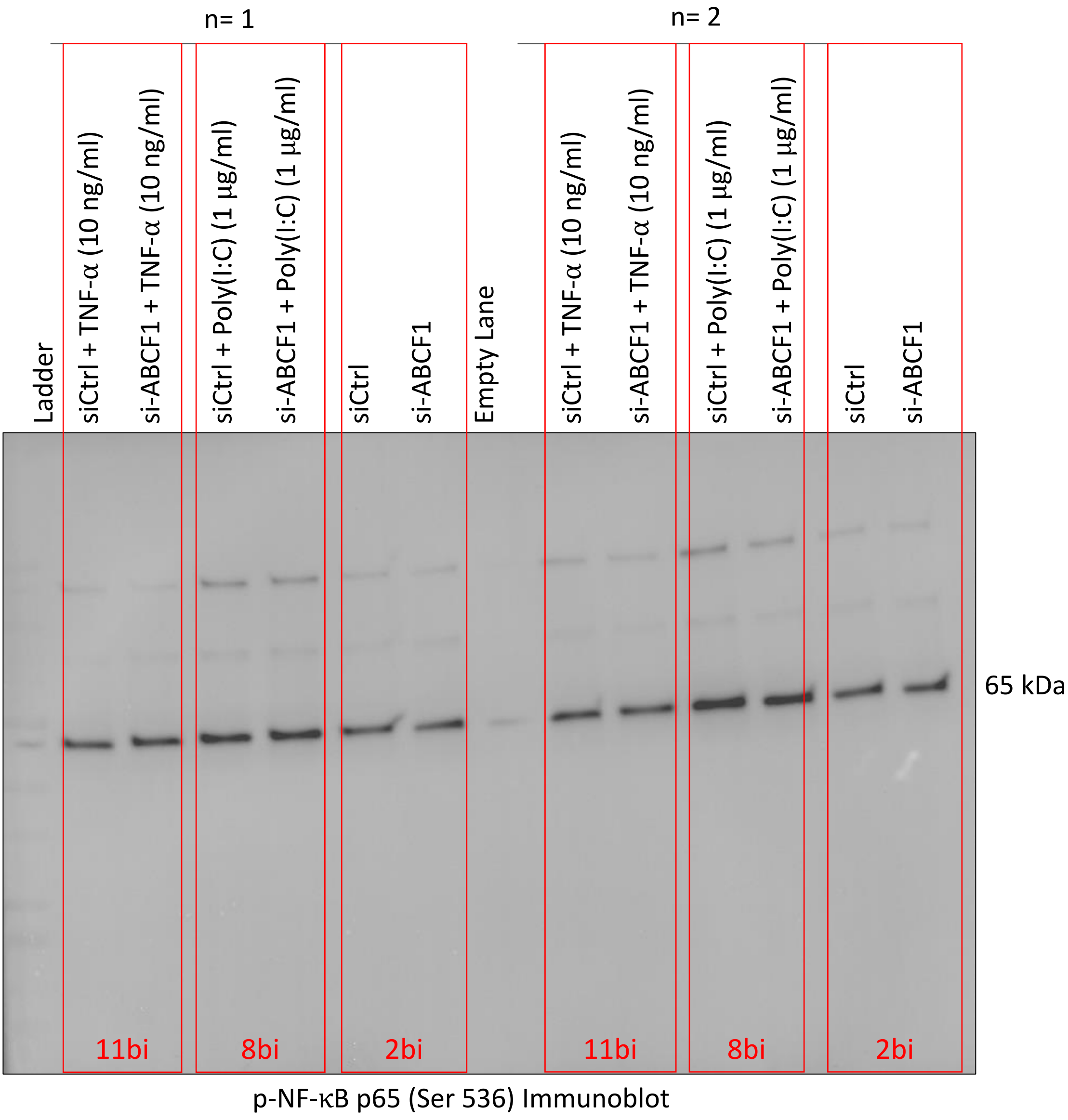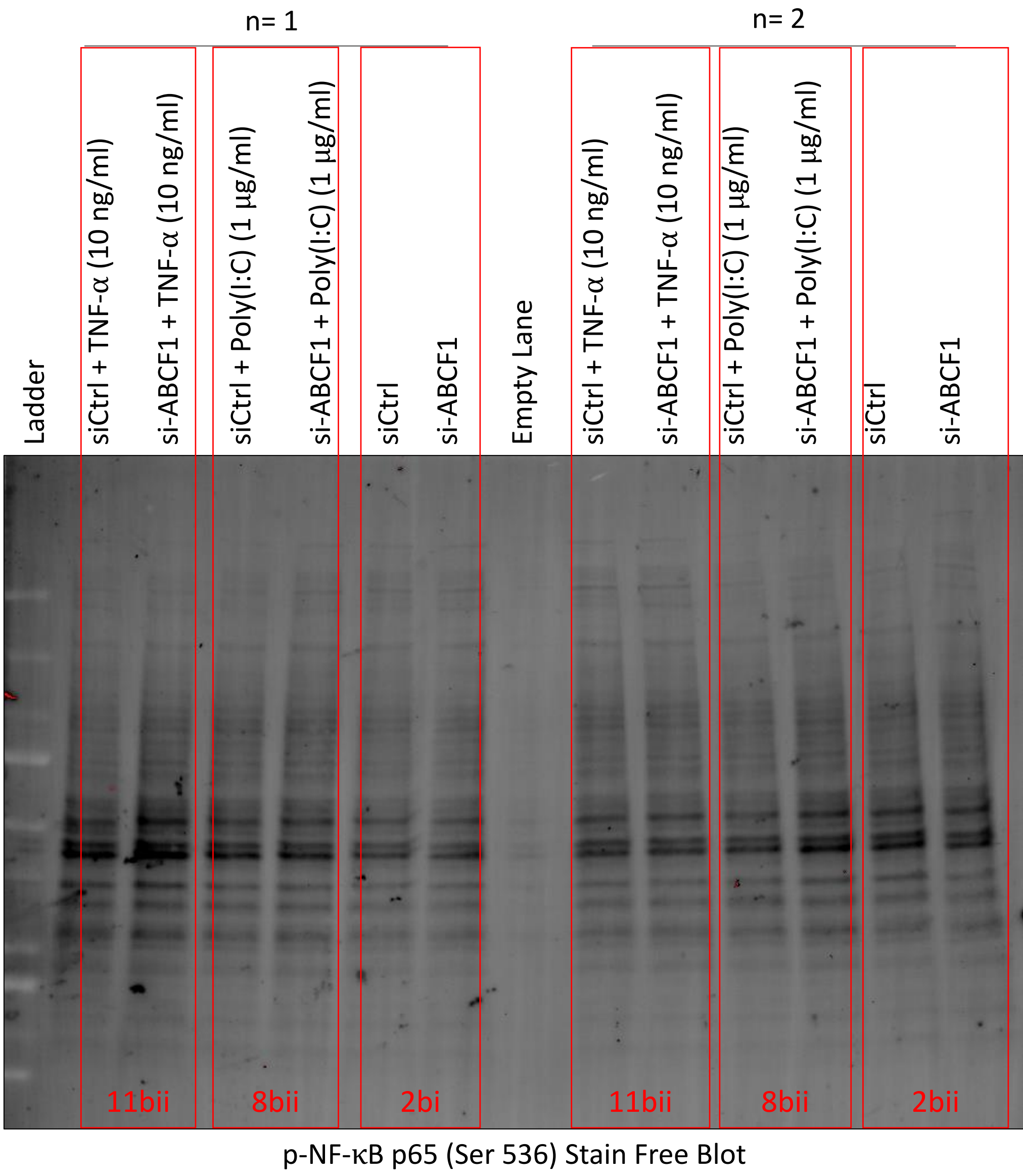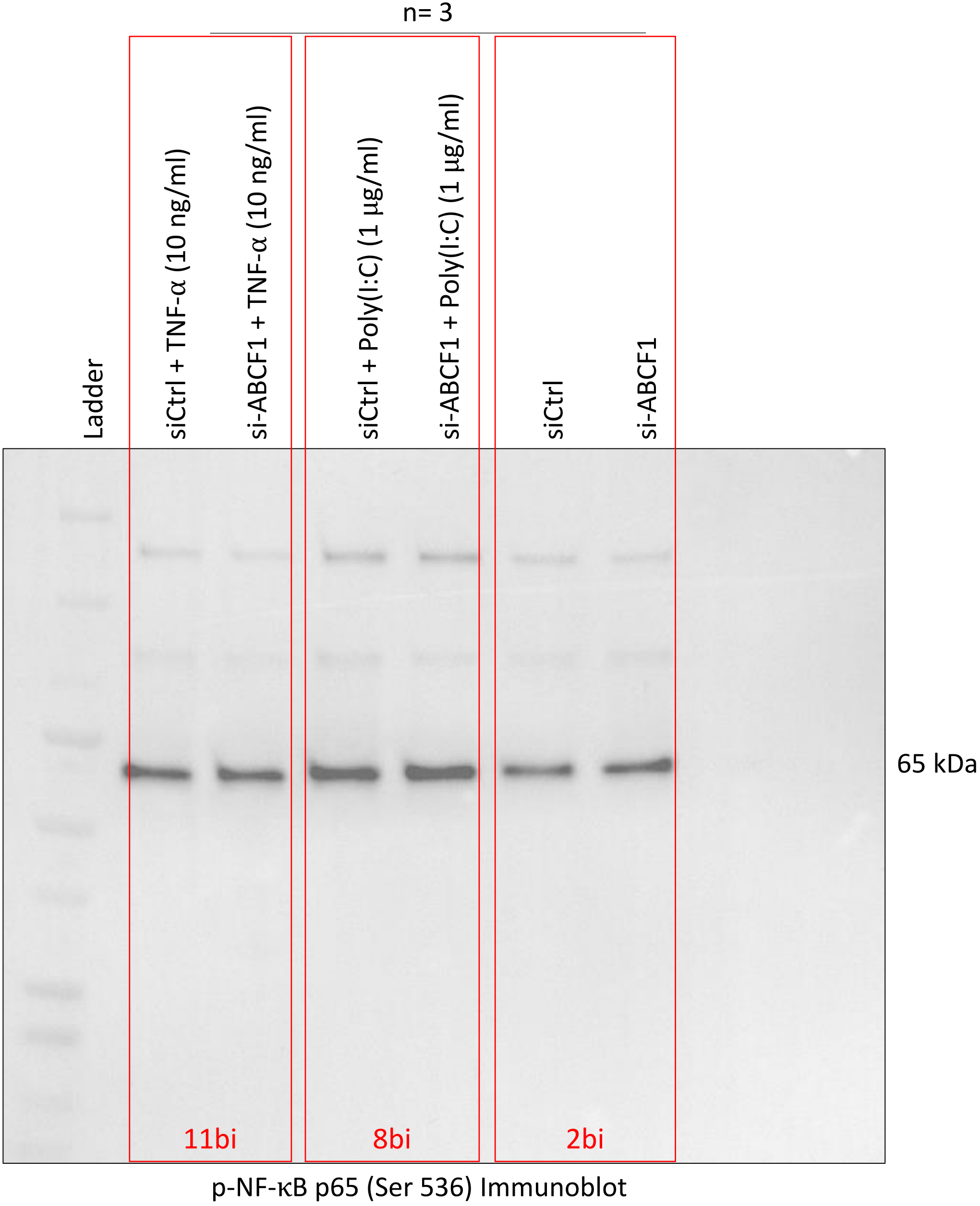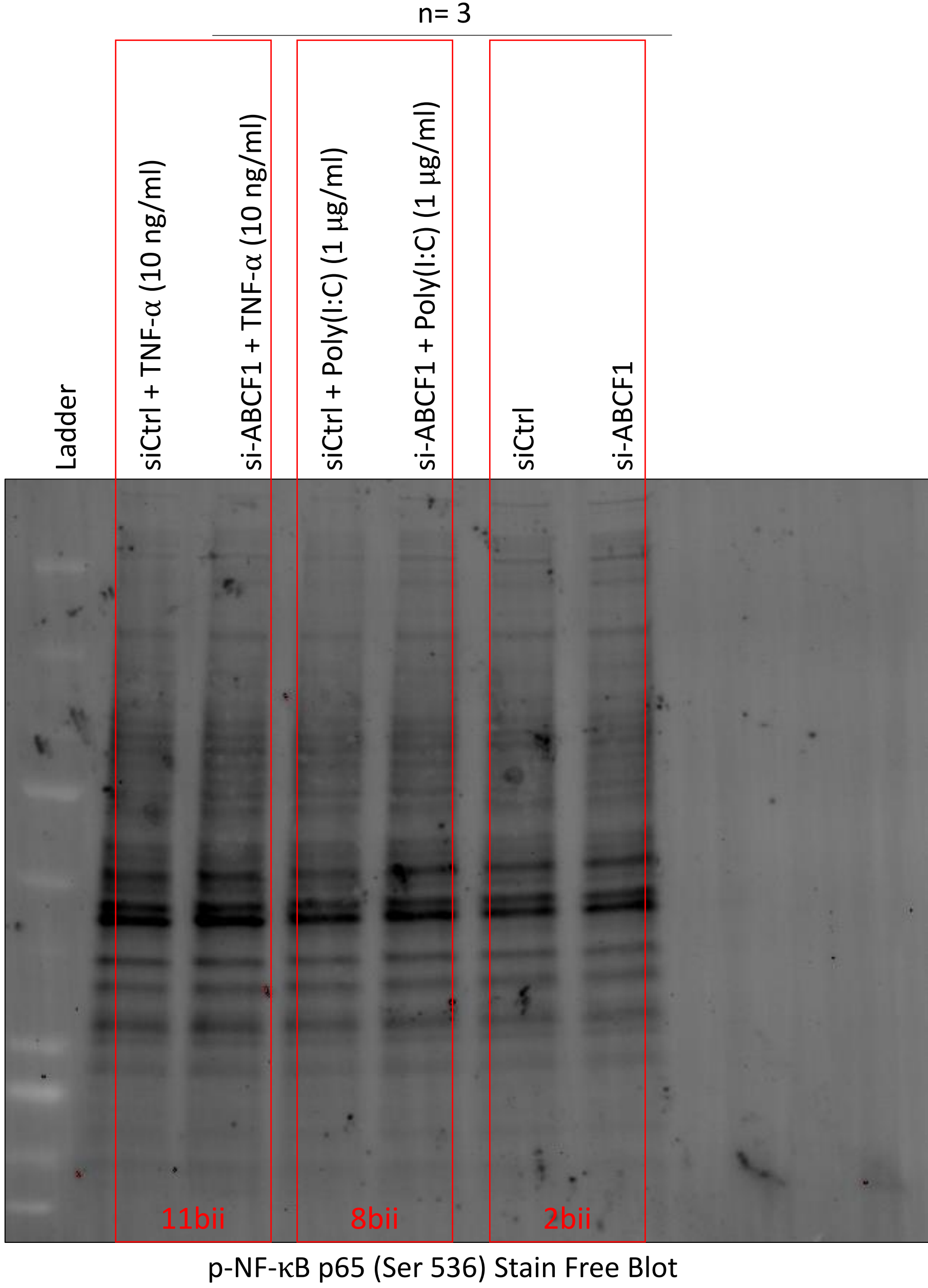

e

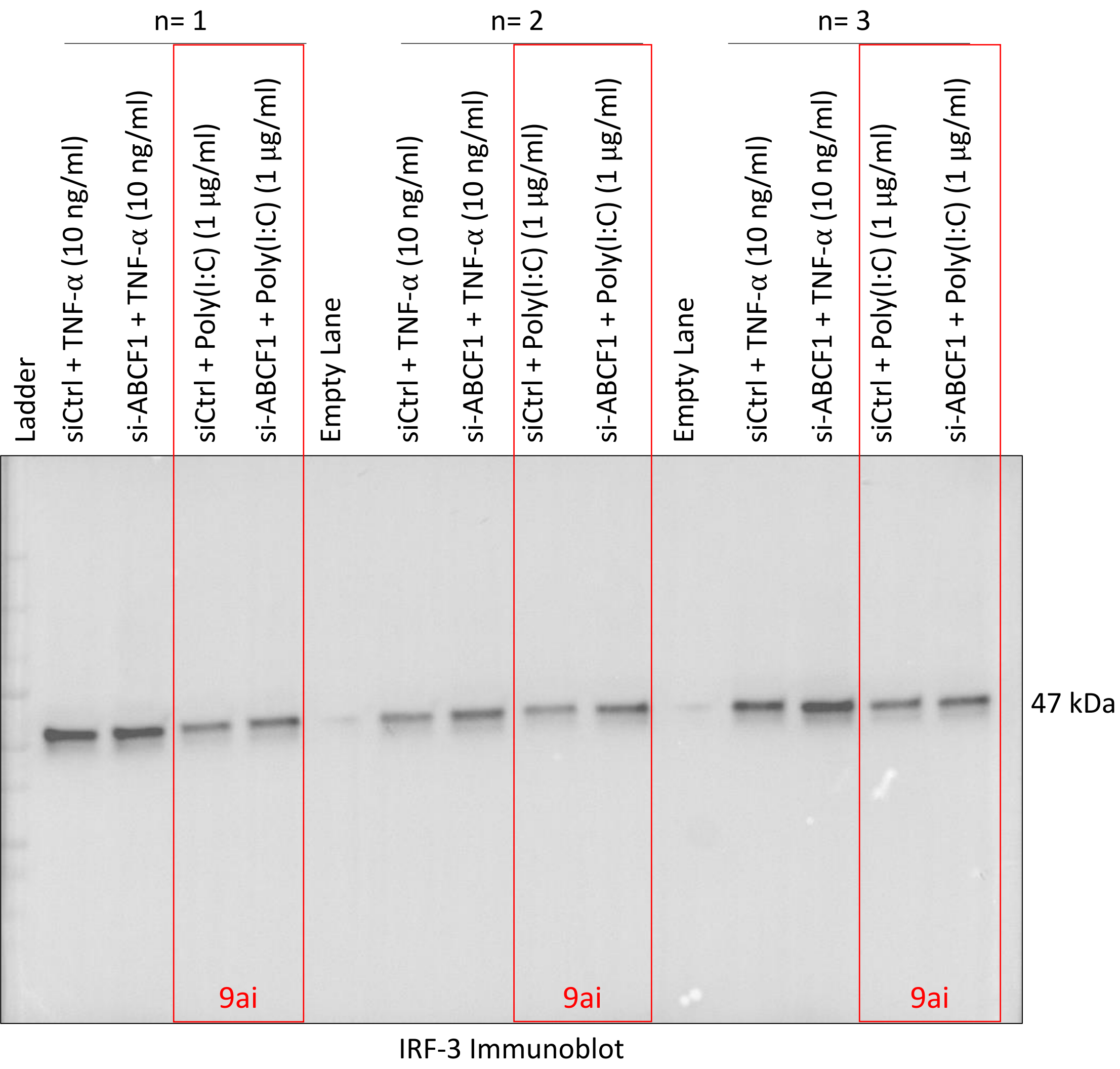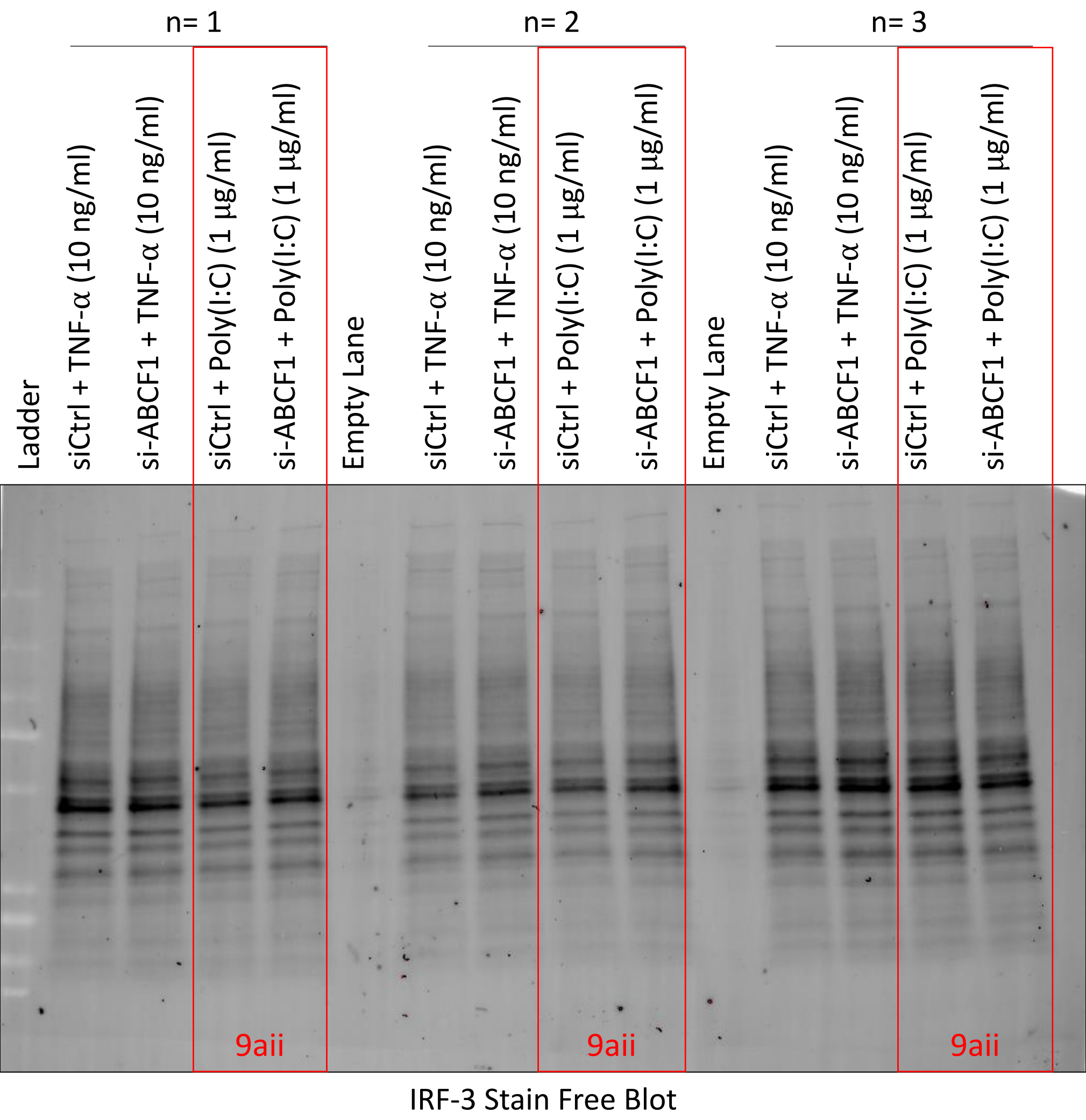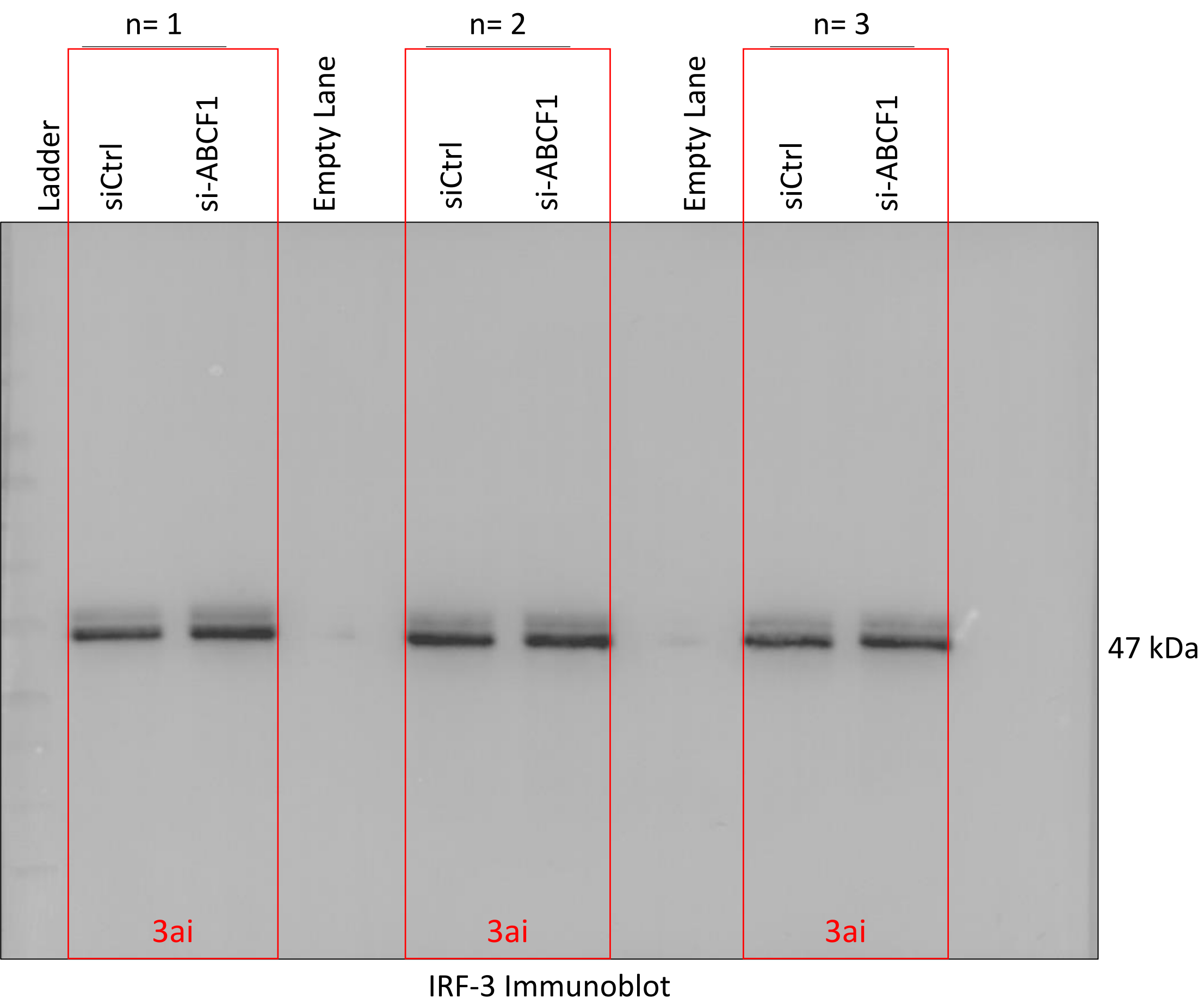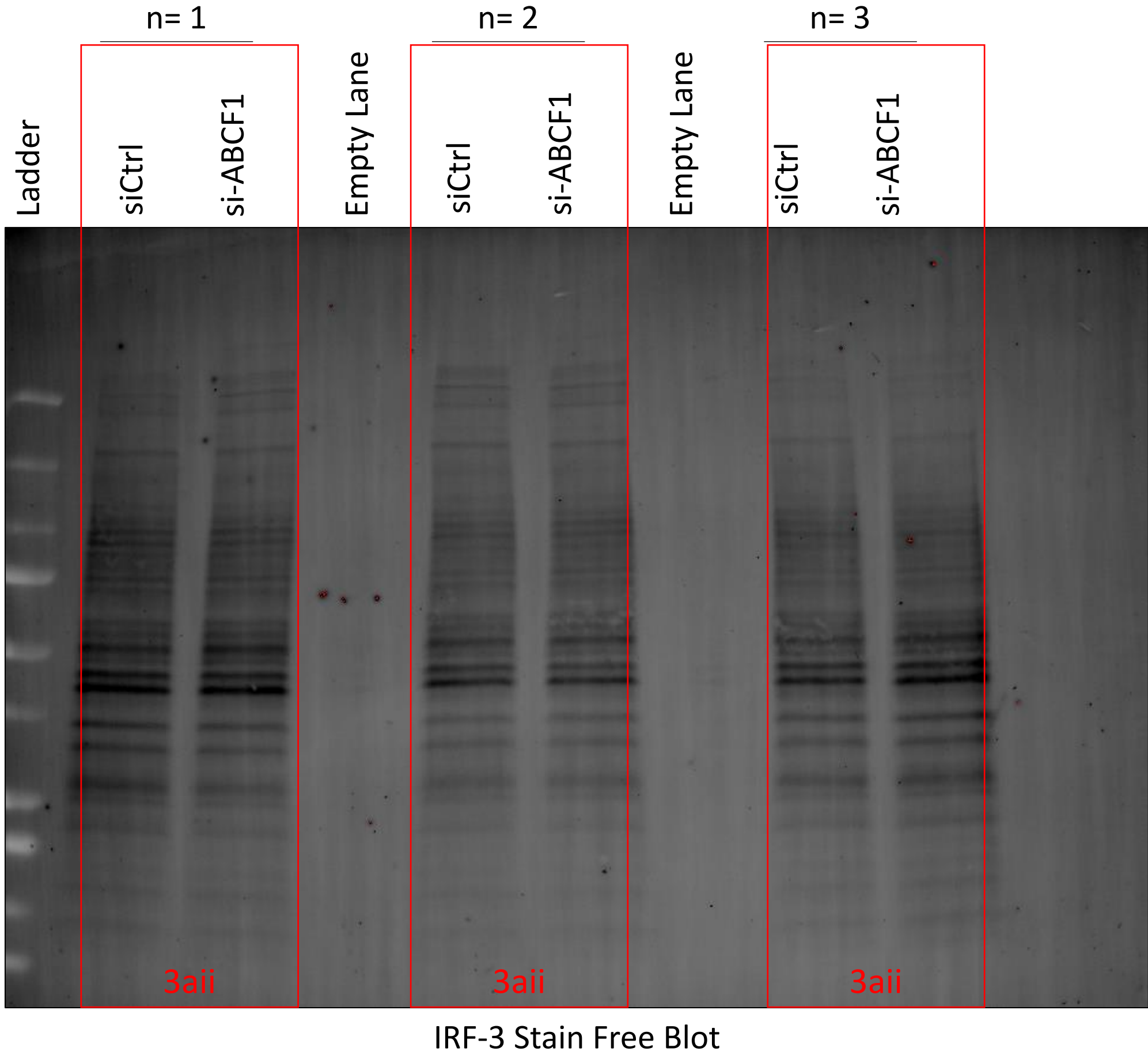

f

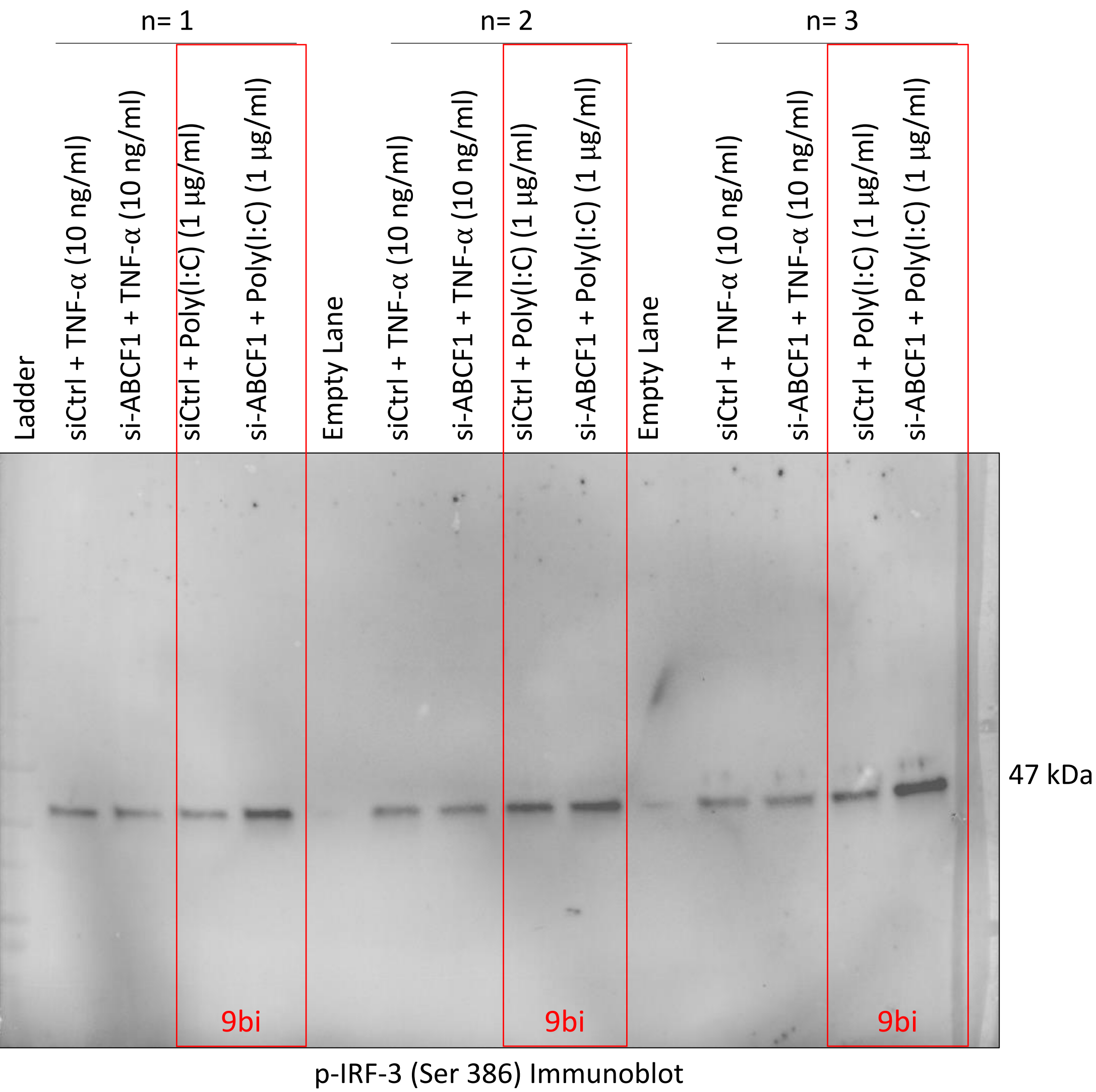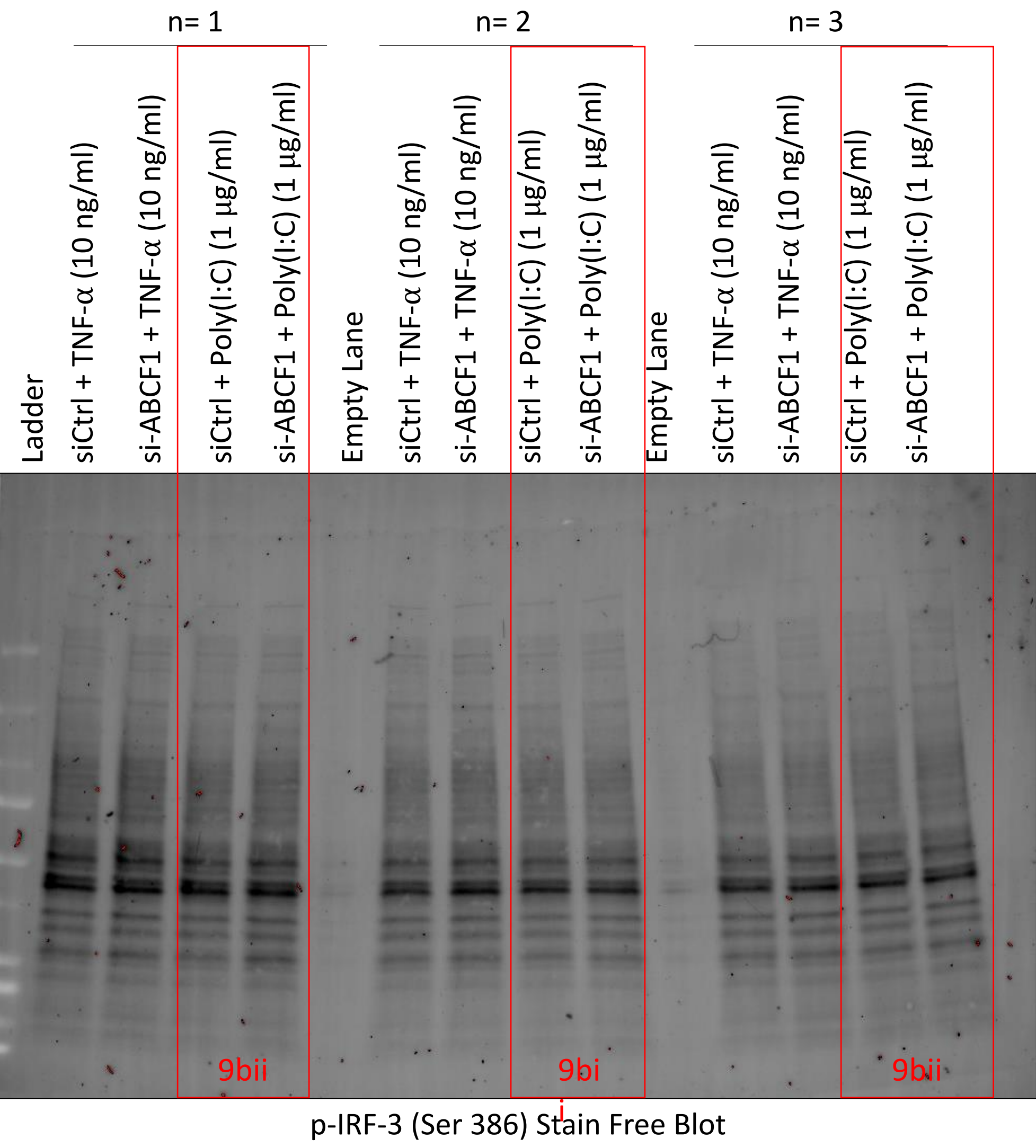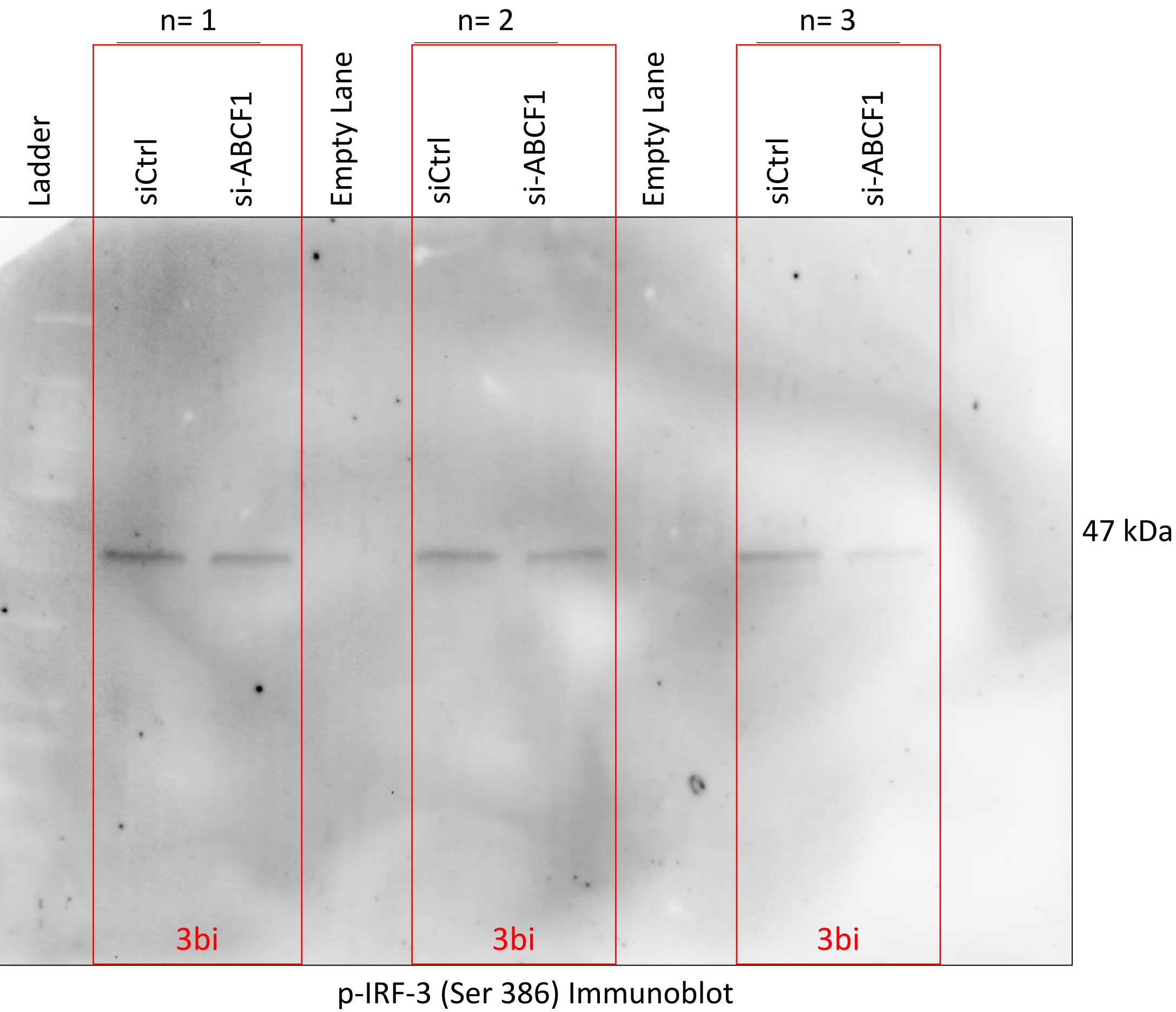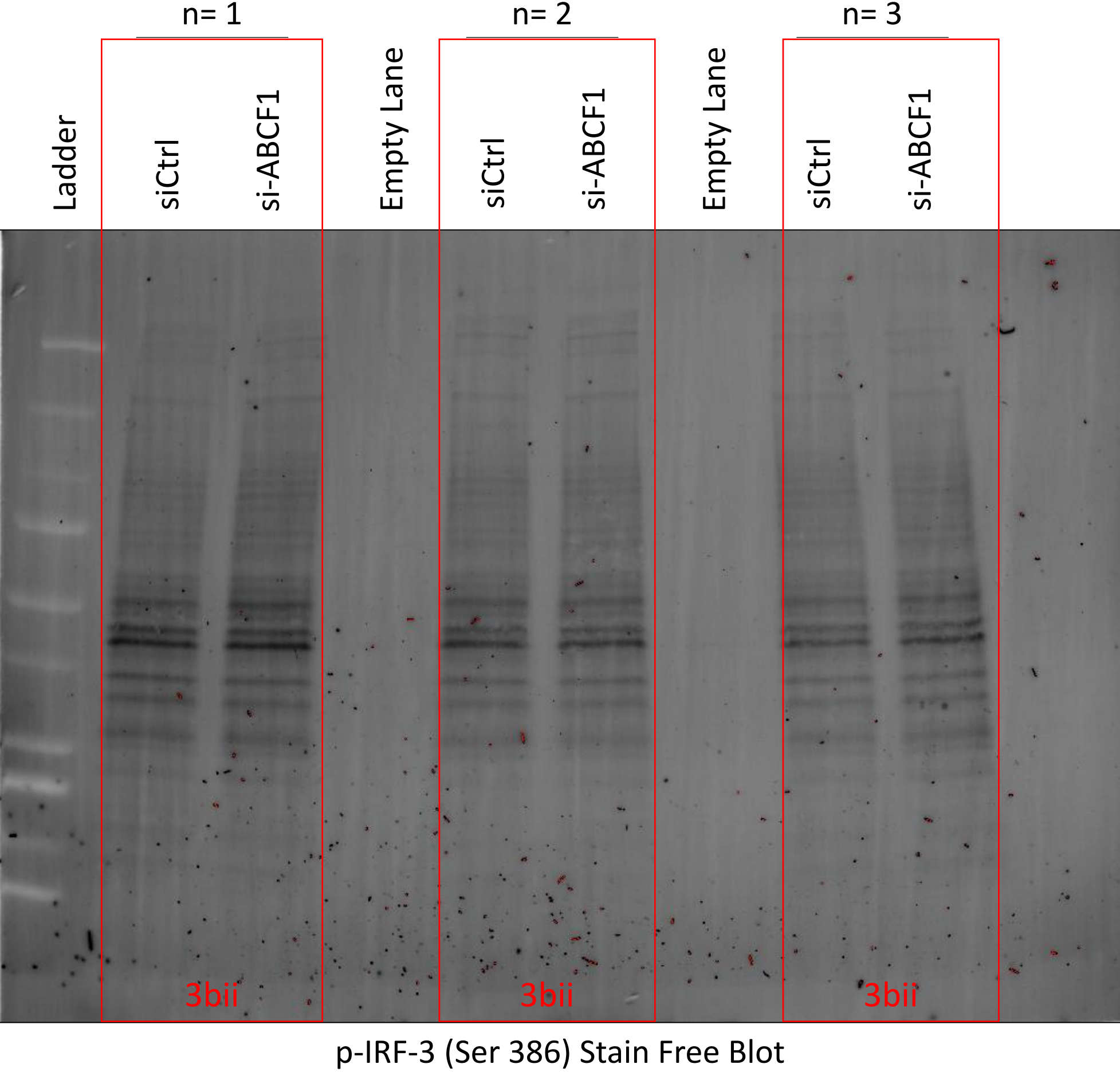

Supplement: Supplementary file 1 — Supplementary Figures. [file 41598_2023_41990_MOESM1_ESM.pdf]
